# THOC5 complexes with DDX5, DDX17, and CDK12 to regulate R loop structures and transcription elongation rate

**DOI:** 10.1016/j.isci.2022.105784

**Published:** 2022-12-09

**Authors:** Mareike Polenkowski, Aldrige Bernardus Allister, Sebastian Burbano de Lara, Andrew Pierce, Bethany Geary, Omar El Bounkari, Lutz Wiehlmann, Andrea Hoffmann, Anthony D. Whetton, Teruko Tamura, Doan Duy Hai Tran

**Affiliations:** 1 Department of Gastroenterology, Hepatology and Endocrinology, Hannover Medical School, Hannover D-30623, Germany; 2 German Cancer Research Center (DKFZ), 69120 Heidelberg, Germany; 3 Stem Cell and Leukemia Protoemics Laboratory, University of Manchester, Manchester M20 3LJ, UK; 4 Institute for Stroke and Dementia Research, Ludwig-Maximilians-Universität, 81377 Munich, Germany; 5 Pädiatrische Pneumologie Hannover Medical School, Hannover D-30623, Germany; 6 Department of Orthopedic Surgery, Hannover Medical School, Hannover D-30623, Germany; 7 Stoller Biomarker Discovery Centre, University of Manchester, Manchester M13 9PL, UK; 8 Institut für Zellbiochemie, Medizinische Hochschule Hannover, Hannover D-30623, Germany; 9 Institut für Humangenetik, Medizinische Hochschule Hannover, Hannover D-30623, Germany

**Keywords:** Molecular biology, Cell biology

## Abstract

THOC5, a member of the THO complex, is essential for the 3′processing of some inducible genes, the export of a subset of mRNAs and stem cell survival. Here we show that THOC5 depletion results in altered 3′cleavage of >50% of mRNAs and changes in RNA polymerase II binding across genes. THOC5 is recruited close to high-density polymerase II sites, suggesting that THOC5 is involved in transcriptional elongation. Indeed, measurement of elongation rates *in vivo* demonstrated decreased rates in THOC5-depleted cells. Furthermore, THOC5 is preferentially recruited to its target genes in slow polymerase II cells compared with fast polymerase II cells. Importantly chromatin-associated THOC5 interacts with CDK12 (a modulator of transcription elongation) and RNA helicases DDX5, DDX17, and THOC6 only in slow polymerase II cells. The CDK12/THOC5 interaction promotes CDK12 recruitment to R-loops in a THOC6-dependent manner. These data demonstrate a novel function of THOC5 in transcription elongation.

## Highlights

• THOC5 depletion decreased transcription elongation rates globally
• 3′ end cleavage of more than 50% of genes was altered in THOC5-depleted cells
• The rate of transcription elongation determines polyA site usage
• THOC5/THOC6 recruit CDK12 and DDX5/17 to regulate R loop structures and elongation rate

## Graphical abstract

**Figure:**
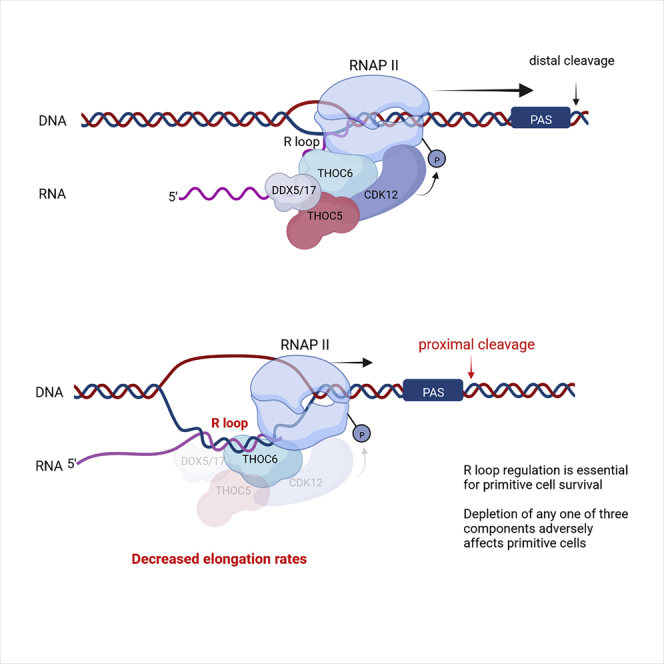

## Introduction

The THO complex, which is a sub-member of TREX (transcription/export) complex, was originally identified in *Saccharomyces cerevisiae* as a five-protein complex (Tho2p, Hpr1p, Mft1p, Thp2p, and Tex1)^1^^,^^2^^,^^3^ that plays a role in transcriptional elongation, nuclear RNA export, and genome stability. In higher eukaryotes such as *Drosophila melanogaster*^4^ or humans,^5^ three equivalent proteins (THOC1/hHpr1, THOC2/hRlr1, and THOC3) and three additional unique proteins were identified, namely THOC5/Fms interacting protein (FMIP),^6^ THOC6, and THOC7, as members of the THO complex.

The THO complex controls the 3′processing of RNA, inhibiting R-loop formation and export of a subset of mRNAs; however, the molecular functions of individual members of this complex are still unclear. Recent data suggest that each member of the TREX complex plays different roles during transcription/export of mRNA.^4^^,^^7^^,^^8^^,^^9^^,^^10^ Evidence indicating that R-loops are a source of genome instability was first provided in yeast cells lacking the THO complex.^11^ In the human system it has been clearly shown that UAP56 (DDX39B), a member of TREX, plays a key role for unwinding harmful R-loops genome wide under DNA damage or replication stress conditions.^10^ Although all members of the THO-complex are detected in the nuclear speckles, Chi and his colleges showed that depletion of THOC1, THOC2, and THOC7, but not THOC5 and THOC6, causes accumulation of polyA + mRNAs in nuclear speckles, indicating that THOC1, THOC2, and THOC7 play a role in mRNA transfer from nuclear speckles to nuclear pores.^12^ These data imply that the THO complex is not one functional unit as previously described.

THOC5 plays a key role in stem cell and cancer cell biology and has been shown to be post-translationally regulated by stem cell ligands (CXCL12), oxidative stress, and the downstream actions of oncogenes.^6^^,^^13^^,^^14^^,^^15^^,^^16^ Our previous data suggest that THOC5 is essential for the mRNA export of only a small subset of genes.^17^^,^^18^^,^^19^ The selectivity of these mRNAs is unclear but we have shown that THOC5 plays a role in 3′end processing of serum inducible genes.^8^ It is, however, still unclear whether THOC5 only plays a role in 3′end processing of inducible genes or whether THOC5 is generally required for the 3′end processing.

In this study, using RNA sequencing by nanopore technology and 5,6-dichlorobenzimidazole 1-β-D-ribofuranoside (DRB)/transient transcriptome sequencing (TT_chem_-seq), we show that THOC5 influences 3′end processing of 50%–60% of alternatively cleaved mRNAs and also participates in transcription elongation by recruiting CDK12 to RNA polymerase II (Pol II) where R-loops are formed. R loops can be formed during cellular stress and as such targeting molecular pathways that regulate their formation or removal, such as THOC5-/THOC6-mediated actions, could provide new approaches for developing therapies.

## Results

### THOC5 depletion modulates mRNA 3′end cleavage

**Figure 1:**
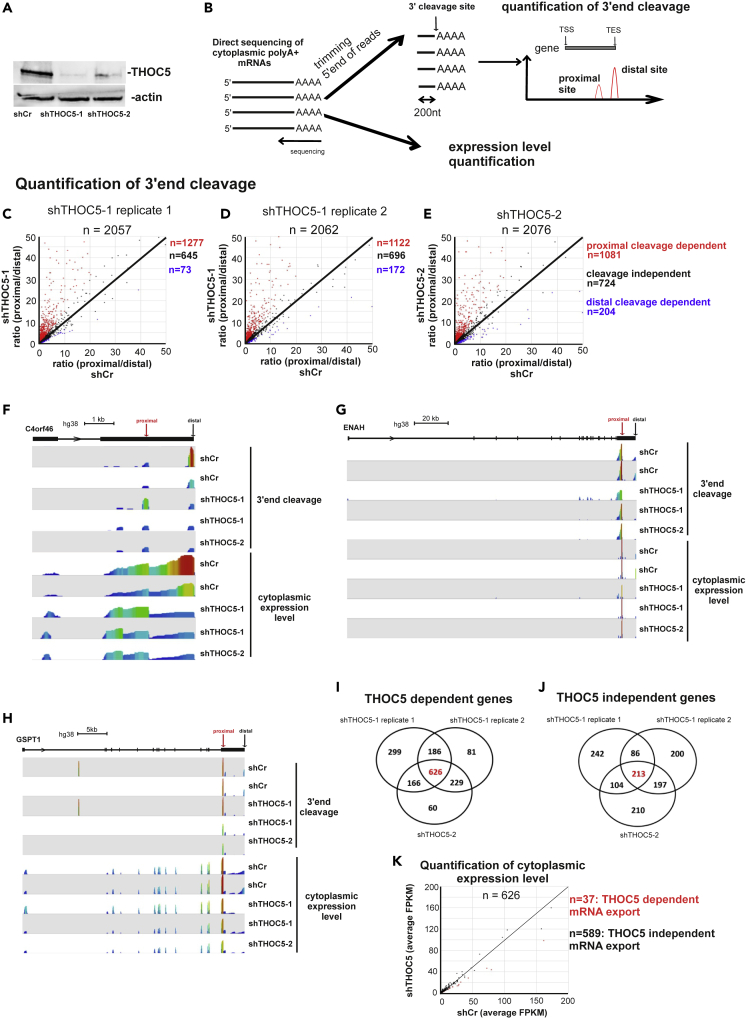
Depletion of THOC5 affects 3′end cleavage of >50% of mRNAs (A) HEK293 cells were transduced with shRNA control (shCr) lentivirus and two different shTHOC5 lentivirus for 4 days. Cells were lysed and THOC5 and actin-specific immunoblotting performed. (B) Cytoplasmic polyA + mRNAs from a sister culture of (A) were isolated and subjected to direct RNA-seq. To map and quantitate 3′end cleavage sites, raw Nanopore reads were trimmed to the last 200 nucleotides from the 3′ end. The 3′end cleavage site usage at the distal and the proximal site were quantitated based on the human poly A database. To analyze mRNA export, full-length Nanopore reads were aligned to hg38, and quantification of cytoplasmic expression level was performed using Seqmonk. (C–E) Transcripts that had at least 5 reads at the distal site were selected to calculate the ratio of proximal to distal site usage. The ratio (proximal/distal) in THOC5-depleted and control cells was plotted. Red dot: proximal cleavage THOC5-dependent genes, black dots: THOC5 independent genes, blue dot: distal cleavage THOC5-dependent genes. (F–H) Wiggle plot of THOC5 proximal cleavage dependent genes *C4ORF46*, *ENAH*, and *GSPT1* gene: Seqmonk was used to quantitate and visualize sequencing data. Peaks in the wiggle plot represent the normalized RNA-seq read coverage. TSS, transcription start site; TES, transcription end site. (I and J) Venn diagram of proximal cleavage THOC5-dependent (I) and independent (J) genes from three experiments were shown. (K) The average read coverages of 626 THOC5-dependent genes in the THOC5-depleted and control cells were calculated using Seqmonk and plotted. See also Figure S1 and Table S1.

We have previously shown that THOC5 plays a role in 3′end processing of several inducible genes.^8^ To examine whether THOC5 level plays a role in 3′processing of only inducible genes or is correlated with alternative cleavage at 3′end of the proximal site and export in the whole genome, we performed cytoplasmic mRNA sequencing using nanopore technology in THOC5-depleted and control cells. Because the depletion of THOC5 induces rapid apoptosis in stem cells,^13^^,^^20^ we utilized HEK293 cells to elucidate its biochemical function. Further, because the complete knockdown of THOC5 has major deleterious effects on cells, we chose depletion as a means of examining function. HEK293 cells were transduced with lentivirus carrying two different short hairpin RNA targeting THOC5 (shTHOC5-1 and shTHOC5-2) and nonsense (shCr) for 4 days. Then cytoplasmic polyA + RNAs were isolated and subjected to direct-RNA sequencing. Four days after infection the downregulation of THOC5 was confirmed by THOC5-specific immunoblot (Figure 1A).

3’end cleavage analysis is depicted in Figure 1B. We selected genes that contained more than two annotated polyadenylation sites (PAS) with more than 5 reads at the distal PAS site. We first determined the ratio between proximal and distal cleavage sites for these genes. Upon depletion of THOC5 we observed an increase of 1.5-fold in the use of proximal cleavage sites in 52%–62% of genes in three independent experiments (Figures 1C, 1D, 1E, red dots), whereas only 3%–10% of genes show an increased distal cleavage (blue dots). The read coverages of three THOC5 proximal-cleavage-dependent genes (Figures 1F–1H), one THOC5 distal-cleavage-dependent gene (Figure S1A), and one THOC5 independent gene (Figure S1B) are shown as an example. Because THOC5 depletion mainly affects the proximal cleavage, we further focused on THOC5 proximal cleavage dependent genes. To identify THOC5-dependent genes we overlapped THOC5 proximal cleavage dependent genes from three independent experiments (Figures 1C–1E). Similar analysis was performed to identify THOC5 independent genes. Six hundred twenty-six THOC5-dependent genes and 213 THOC5 independent genes were identified (Figures 1I and 1J; Table S1). We then examined the nuclear mRNA export of three THOC5-dependent genes by analyzing nuclear and cytoplasmic RNA level. Interestingly, the mRNA export of these genes was not altered upon THOC5 depletion (Figure S1C). Thus, we further quantified the cytoplasmic RNA level of THOC5-dependent mRNAs from Nanopore RNA-seq data. In agreement with mRNA export assay data (Figure S1C), cytoplasmic RNA level of only 37 genes were reduced more than 1.5-fold, suggesting that most of the mRNAs cleaved at the proximal site were exported equally well (Figure 1K).

### THOC5 influences Pol II transcription elongation

**Figure 2:**
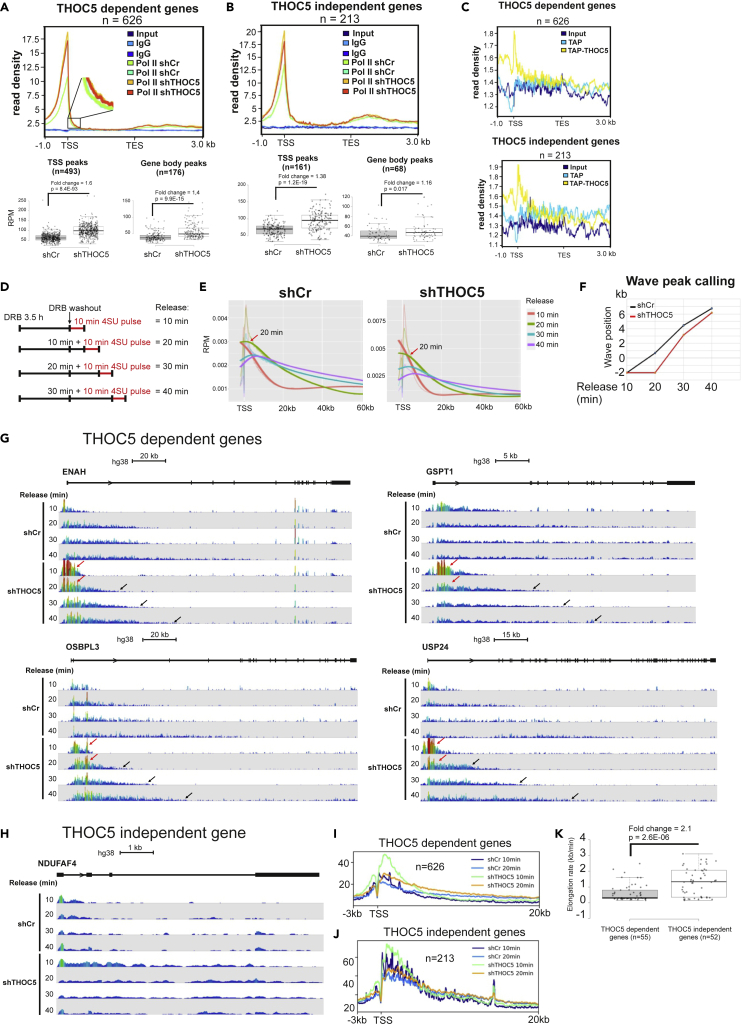
THOC5 influences on transcription elongation (A and B) Metaplot of mean Pol II ChIP signals for 626 THOC5-dependent (A) and 213 THOC5 independent (B) genes were generated using deepTools2.^21^ 50-base bins are shown in flanking regions from 1 kb relative to the TSS and from 3 kb relative to the polyA site. Input and IgG were used as negative control. MACS peak caller module of Seqmonk (p value cut-off = 10^−5^) was applied to identify and quantify peaks at the TSS and gene body. Read density of peaks was normalized to sequencing depth. p: p value. (C) Metaplot of mean TAP-THOC5 ChIP signals for 626 THOC5-dependent and 213 THOC5 independent genes. (D) Outline of DRB inhibition and 4SU labeling times used for DRB/TTchem-seq. (E) DRB/TTchem-seq metagene profiles of protein-encoding genes between 60 and 300 kb from standard chromosomes (1–22, X, Y) with non-overlapping transcriptional units (n = 4591). Lines are computationally fitted splines. (F) Calculation of Pol II transcription wave position based on metagene profiles using linear regression. (G and H) Coverage profiles of DRB/TT-seq results for THOC5-dependent (I) and THOC5 independent (J) genes. Black arrows indicated the delayed elongation rates upon THOC5 depletion. Red arrows indicated accumulations of Pol II molecules short after TSS. (I and J) DRB/TTchem-seq metagene profiles of 626 THOC5-dependent and 213 THOC5 independent genes. (K) Elongation rates of THOC5-dependent and independent genes were plotted. See also Figure S2 and Table S2.

To examine whether THOC5 influences RNA Pol II pausing at the 3′end, we performed Pol II ChIP sequencing (ChIP-Seq) in THOC5-depleted and control cells. In parallel, we performed tandem affinity purification (TAP)-THOC5 CHIP sequencing. Upon depletion of THOC5, Pol II density clearly increases at the TSS site, whereas only a slight increase was observed in gene body and at the 3′end of 626 THOC5-dependent genes (Figure 2A). We then quantified Pol II ChIP-seq peaks located at the TSS and gene body using MACS peak calling^22^ (p value cut-off = 10^−5^). Four hundred ninety-three TSS and 176 gene body peaks were detected. Upon THOC5 depletion the average peak intensity significantly increased more than 1.4-fold at both TSS (p = 8.4E-93) and gene body (9.9E-15) of THOC5-dependent genes. The depletion of THOC5 also induced a Pol II accumulation of 1.4-fold at the TSS of THOC5 independent genes (Figure 2B, p = 1.2E-19), whereas the average peak intensity at the gene body of these genes only slightly increased (1.1-fold, p = 0.017). Notably, TAP-THOC5 is mainly recruited to the promoter and shortly after the TSS and moderately recruited to the gene body of THOC5-dependent genes, whereas the recruitment to the gene body was not observed for THOC5 independent genes (Figure 2C), suggesting that THOC5 may play a role in transcription elongation. To examine this point, we performed DRB/TTchem-seq, which combines transient transcriptome sequencing (TT-seq) with transient inhibition of early elongation using the reversible CDK9 inhibitor, 5,6-dichlorobenzimidazole 1-β-D-ribofuranoside (DRB), to measure RNA polymerase II (RNAPII) elongation rates *in vivo* in control and THOC5-depleted cells.^23^ The protocol is depicted in Figure 2D. The incorporation of 4SU (4-thiouridine) was tested (Figures S2A and S2B). We kept the 4SU-labeling time constant at 10 min to avoid any bias due to the difference in 4SU treatment. To measure the progression of Pol II molecules into the gene body, we applied a pipeline described by Gregersen et al.^23^ for calling RNA Pol II transcription wave peak positions and elongation rates from DRB/TT-seq time-series data. The pipeline first created a set of genomic intervals representing the TSS region (−2 kb: +120 kb) of non-overlapping protein coding genes 60–300 kb in width from standard chromosomes (4591 genes). Ten minutes after DRB release, most Pol II molecules were synchronized shortly after the TSS in control and THOC5-depleted cells (Figures 2E and 2G); 20, 30, and 40 min after DRB release, the bulk of released RNAPII molecules have moved downstream of the TSS in control cells, whereas the progression of Pol II was delayed in THOC5-depleted cells (Figure 2E, red arrow). We then calculated the wave peak using wave peak calling function. THOC5 depletion clearly delayed the wave peaks of the Pol II, suggesting that THOC5 plays a role in transcription elongation (Figure 2F). The read coverage of THOC5-dependent genes also show a delayed Pol II progression (Figure 2G, black arrows), whereas the delay was not observed for *NDUFAF4*, a THOC5 independent gene (Figure 2H). In addition, we observed an accumulation of Pol II molecules shortly after the TSS of THOC5-dependent genes after 10- and 20-min DRB release in THOC5-depleted cells (Figure 2G, red arrows). Metagene analysis for 626 THOC5-dependent and 213 THOC5 independent genes also revealed a clear accumulation upon THOC5 depletion short after the TTS of THOC5-dependent genes (Figure 2I) but not that of THOC5 independent genes (Figure 2J). These data also agreed with Pol II ChIP-seq data in that Pol II molecules are accumulated short after the TSS in THOC5-depleted cells (Figure 2A), suggesting that THOC5 depletion caused an accumulation of Pol II near to the TSS and delayed transcription elongation. These data raised the question of how THOC5 selects its target genes? Thus, we applied the module “wave peak calling for single gene” to calculate the elongation rates and examine the alternative 3′ cleavage of these genes. Elongation rates of 226 highly expressed non-overlapping protein coding genes 60–300 kb in width were calculated using this module. Among those genes, mRNAs of 107 genes underwent alternative 3′cleavage detected by Nanopore-seq. Notably, THOC5 depletion increased the proximal cleavage of 55 mRNAs in at least two out three independent experiments (Table S2). We then plotted elongation rates of THOC5-dependent and independent genes. As shown in Figure 2K, the average elongation rate of THOC5-dependent genes was 2.1-fold lower than that of THOC5 independent genes (p = 2.6E-06), suggesting that THOC5 may target genes with slow elongation rates. These data raise the question of whether changes of elongation rates influence 3′end cleavage.

### Changes of elongation rates influence polyA site choice

**Figure 3:**
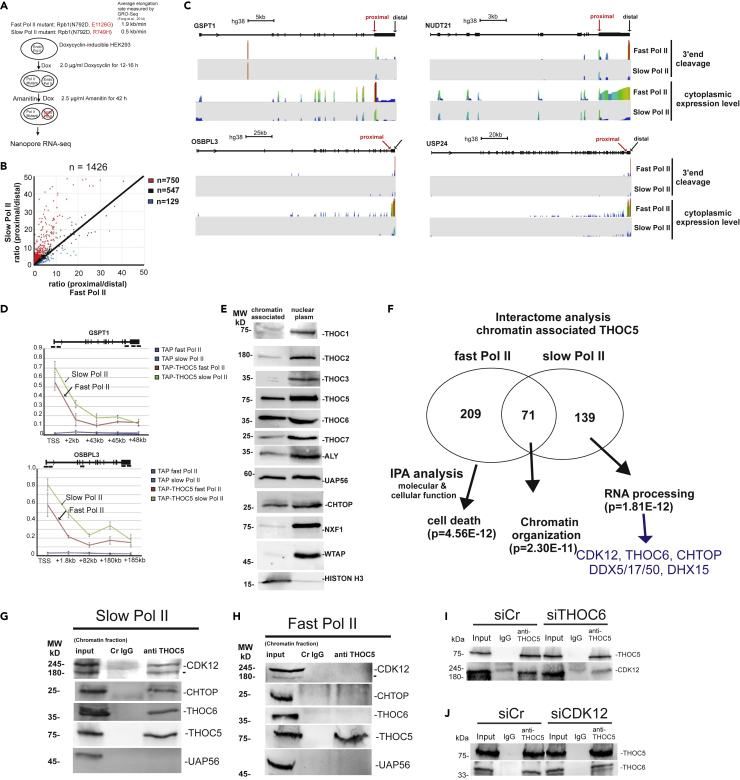
Chromatin-associated THOC5 interacts with CDK12 in slow Pol II cells but not in fast Pol II cells and influences CDK12 recruitment during transcription elongation (A) Fast and slow Pol II mutants were induced by Doxycycline treatment. Cytoplasmic RNAs were isolated and subjected to Nanopore direct RNA-seq. (B) The ratio (proximal/distal) in fast and slow Pol II cells was plotted. Red dot: slow Pol II-dependent proximal cleavage genes, black dots: slow Pol II independent genes, blue dot: slow Pol II-dependent distal cleavage genes. (C) Wiggle plot of *GSPT1*, *OSBPL3*, *NUDT21*, and *USP24* gene: Seqmonk was used to quantitate and visualize sequencing data. Peaks in the wiggle plot represent the normalized RNA-seq read coverage. TSS: transcription start site. TES: transcription end site. (D) pNTAP (TAP) and pNTAP carrying THOC5 cDNA (TAP-THOC5) were transfected into fast and slow Pol II cells for 24 h. After cross-linking by adding formaldehyde, protein and DNA were extracted and the chromatin was sheared by sonication. Cell extracts and binding fractions with streptavidin Sepharose were analyzed by *GSPT1* (TSS, +2 kb, +43 kb, +45 , and 48 kb) and *OSBPL3* (TSS, 1.8 , 80 , 180 , and 185 kb). (E) Chromatin-associated and nucleoplasmic fractions of HEK293 cells subjected to immunoblot for TREX complex components and NXF1. WTAP and histone H3 were used as markers for chromatin-associated and nucleoplasmic fractions. (F) Venn diagram showing the overlap of proteins identified by the chromatin-associated THOC5 interactome analysis in fast and slow Pol II cells. IPA analysis was performed to identify any functional enrichment observed within the proteins within each sub-group. The significance (p values) for the function enrichment was calculated using the right-tailed Fisher exact test.^25^ (G and H) Interaction between chromatin-associated THOC5 and CDK12, CHTOP, and THOC6 in slow Pol II cells (G) was confirmed by endogenous THOC5 co-IP study in slow Pol II cells. Fast Pol II cells were used as a negative control (H). Chromatin-associated fractions were isolated and supplied for THOC5 co-IP assay. (I) Interaction between chromatin-associated THOC5 and CDK12 in THOC6-depleted cells was examined by endogenous THOC5 co-IP study. (J) Interaction between chromatin-associated THOC5 and THOC6 in CDK12-depleted cells was examined by endogenous THOC5 co-IP study. See also Figures S3, S7, Tables S3 and S4.

To examine whether changes of elongation rates influence 3′end cleavage, we utilized inducible cell lines expressing α-amanitin-resistant mutants of the human Pol II large subunit that accelerate or decelerate elongation.^24^ Then we performed Nanopore RNA-seq for cytoplasmic RNAs isolated from cells expressing fast or slow Pol II (kind gift from David Bentley) (Figure 3A) to determine the ratio between proximal and distal cleavage sites of genes that contained more than two annotated polyadenylation sites (PAS) with more than 5 reads at the distal PAS site in cells expressing fast Pol II (1426 genes). Seven hundred fifty out of 1,426 genes increased more than 1.5-fold 3′ cleavage at proximal sites in cells expressing slow Pol II (Figure 3B, red dots), whereas an increase in the use of distal cleavage sites was observed in only 129 genes (Figure 3B, blue dots), suggesting that slow Pol II provokes 3′ cleavage at the proximal sites. Notably, polyA site usage of THOC5 target genes is clearly dependent on elongation rates (Figure 3C). This raises the next question of whether THOC5 is more requisite to support transcription elongation in cells expressing slow Pol II. To examine this point, we performed TAP-THOC5 ChIP using cells expressing fast or slow Pol II. TAP-THOC5 is more highly recruited to THOC5-dependent genes in slow Pol II cells compared with fast Pol II cells (Figure 3D), suggesting that cells have a greater requirement for THOC5 when the elongation rate is low.

### Chromatin-associated THOC5 interacts with CDK12 in the presence of slow Pol II, but not fast Pol II

To examine whether all members of TREX participate in transcription elongation and 3′processing, we examined which members of TREX are associated with chromatin. Notably, only THOC5, THOC6, UAP56, and CHTOP were clearly detected in the chromatin-associated fraction at levels comparable to the nucleoplasmatic fraction (Figure 3E). We then performed interactome analysis using chromatin-associated THOC5 as bait in slow and fast Pol II cells; 281 and 211 proteins were detected in fast and slow Pol II expressing cells, respectively (Figure 3F, Table S3). Only 71 proteins were common. Among TREX complex members, only THOC6 and CHTOP were detected with THOC5 on chromatin (Figure 3F). Ingenuity Pathway Analysis (IPA) of the unique and shared binding proteins revealed that the top molecular cellular function category identified in the common binding proteins was “chromatin organization,’’ whereas in the fast Pol II cell-specific proteins it was “cell death,” and in the slow cell-specific binding partners it was “RNA processing” with 20 proteins in this category (Figure 3F, Table S4).

Importantly, one of the proteins unique to the slow Pol II fraction is CDK12, a protein that controls Pol II elongation rate by phosphorylation of the Pol II carboxy-terminal domain (CTD).^26^ To confirm the interaction between THOC5 and CDK12, we performed co-immunoprecipitation using fast and slow Pol II cells. In agreement with interactome analysis data, THOC5 interacts with CDK12 only in slow Pol II cells (Figures 3G, 3H, S3A and S3B). Notably, THOC5 depletion did not alter the protein level of CDK12 (Figure S2C). Among the members of the THO complex, only THOC6 was found to interact with THOC5 in the chromatin fraction (Figures 3G, 3H, S3A and S3B; Table S4). Interestingly, THOC5-CDK12 interaction was drastically reduced in THOC6-depleted cells (Figure 3I), whereas CDK12 depletion did not affect the THOC5-THOC6 interaction (Figure 3J), suggesting THOC5 and THOC6 may form a sub-complex and participate in transcription elongation through recruiting CDK12. Furthermore, DEAD box helicase (DDX) 5, 17, 50, and DEAH box helicase (DHX) 15, which may resolve R-loop formation, also interacted with chromatin-associated THOC5 (Table S4).

### 3′end cleavage of THOC5-dependent genes is regulated by THOC6 and CDK12

**Figure 4:**
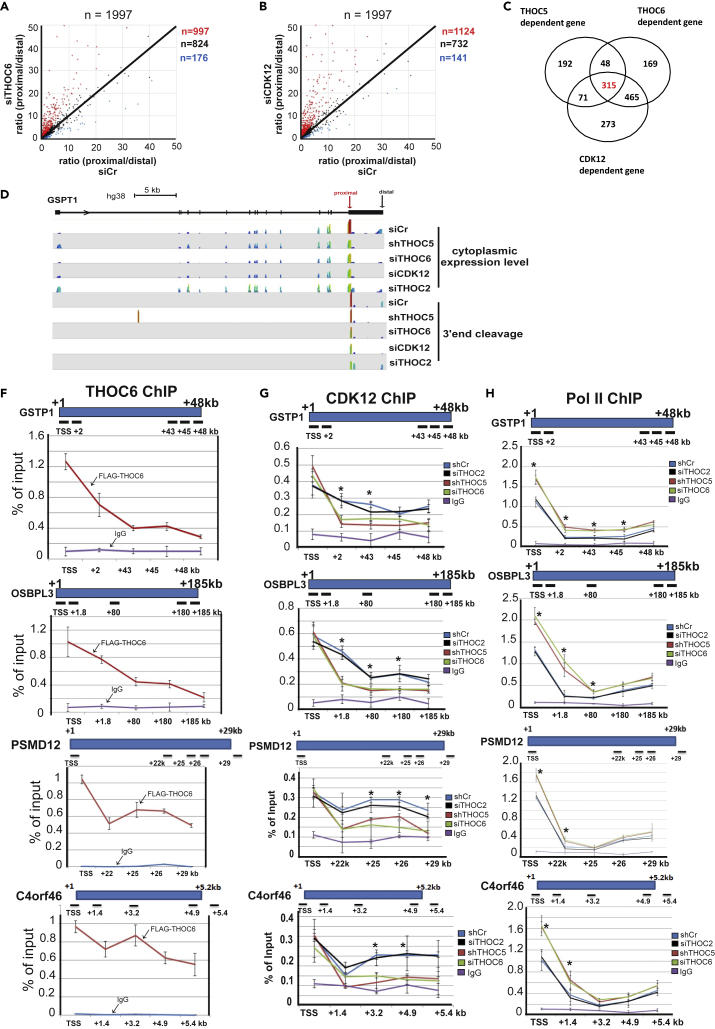
Depletion of THOC6 and CDK12 affects 3′end cleavage of >50% of mRNAs (A and B) HEK293 cells were transfected with siRNA control (siCr) and siTHOC6 or siCDK12 for 3 days. Cytoplasmic polyA + mRNAs were isolated and subjected to direct RNA-seq. The ratio (proximal/distal) in THOC6- (A) and CDK12 (B)-depleted cells was plotted. Red dot: THOC6/CDK12-dependent proximal cleavage genes, black dots: THOC6/CDK12 independent genes, blue dot: THOC6/CDK12-dependent distal cleavage genes. (C) Venn diagram of THOC5-, THOC6-, and CDK12-dependent genes. (D) Wiggle plot of GSPT1 gene: Seqmonk was used to quantitate and visualize sequencing data. Peaks in the wiggle plot represent the normalized RNA-seq read coverage. TSS: transcription start site. TES: transcription end site. (F) FLAG-tagged THOC6 was overexpressed in HEK293 cells. Cells were fixed in 1% (v/v) PFA and supplied for ChIP assay using FLAG M2 mouse monoclonal antibody. Mouse IgG was used as a control. The recruitment profile of THOC6 to *GSPT1*, *OSBPL3*, *PSMD12*, and *C4ORF46* genes is shown. Three independent experiments were performed, and the average signal of ChIP is shown as the mean value +/− SD. (G and H) THOC5 were depleted in HEK293 cells by lentivirus (sh), whereas THOC2 or THOC6 were downregulated by siRNAs. Cells were fixed in 1% PFA and CDK2 and Pol II ChIP assay performed. The recruitment profile of CDK12 (G) and Pol II (H) to *GSPT1*, *OSBPL3*, *PSMD12*, and *C4ORF46* genes in the presence and absence of THOC5 or THOC6 is shown. Three to five independent experiments were performed, and the average signal of ChIP is shown as the mean value +/−SD. ∗p value <0.05. See also Figure S4.

To further examine the connection between THOC5, THOC6, and CDK12, we depleted CDK12 and THOC6 using siRNA and analyzed 3′end cleavage using Nanopore RNA-seq. Similar to THOC5 depletion, more than 50% of mRNAs shifted 3′cleavage to proximal sites in THOC6- or CDK12-depleted cells (Figures 4A and 4B, read dots), whereas less than 10% of mRNA shifted 3′cleavage to distal sites (Figures 4A and 4B, blue dots). Notably, 3′cleavage of 50% (315 genes) of THOC5-dependent genes (626 genes) is also regulated by CDK12 and THOC6 (Figures 4C and 4D). Then we tested whether the THOC5/THOC6 complex is involved in the transcription elongation by performing a-THOC6-FLAG ChIP assay using FLAG antibody. In parallel, we also performed a CDK12- and Pol II ChIP assay in the presence and absence of THOC5 or THOC6. The recruitment of THOC6 and CDK12 to four THOC5-dependent and one THOC5 independent genes (*NDUFAF4*) was examined. Similar to THOC5, THOC6 was found to be recruited in the promoter region and the gene body of THOC5-dependent genes (Figure 4F), whereas the gene body recruitment was less in THOC5 independent gene (Figure S4A). CDK12 has a similar recruitment profile to that of THOC6 in that it is recruited to both the promoter and the gene body (Figure 4G). We next depleted THOC5 and THOC6 (Figure S4B) and examined CDK12 and Pol II recruitment. Upon THOC5 or THOC6 depletion, the recruitment of CDK12 was significantly reduced short after TSS of THOC5 of THOC5-dependent genes, whereas the reduction of CDK12 recruitment to THOC5 independent gene was not significant (Figure S4C). Depletion of THOC5 or THOC6 induced an accumulation of Pol II at the TSS and short after the TSS (Figure 4H). These data suggest that the THOC5-THOC6 complex participates in CDK12 recruitment during transcription elongation and controls 3′end cleavage of a subset of genes.

To examine whether the THOC5/THOC6 complex (but not other members of the THO complex) is involved in control of the elongation rate, we depleted THOC2, a key member of THO complex (Figure S4D). THOC2 is mainly located in nucleoplasm (Figure 3E) and does not interact with THOC5 in the chromatin-associated fraction (Table S4). THOC2 depletion only slightly affected gene body recruitment of CDK12 to THOC5-dependent genes (Figure 4G). Consequently, THOC2 depletion did not influence 3′ cleavage of THOC5-dependent mRNA (Figure 4D). These data suggest that the THOC5/THOC6 complex participates in the transcriptional elongation rate by recruiting CDK12. Notably, the change of elongation rate directly affects 3′ cleavage. These facts raise the next question of how the THOC5/THOC6 complex is recruited during transcription elongation.

### THOC5/THOC6 depletion induces R-loop accumulation

**Figure 5:**
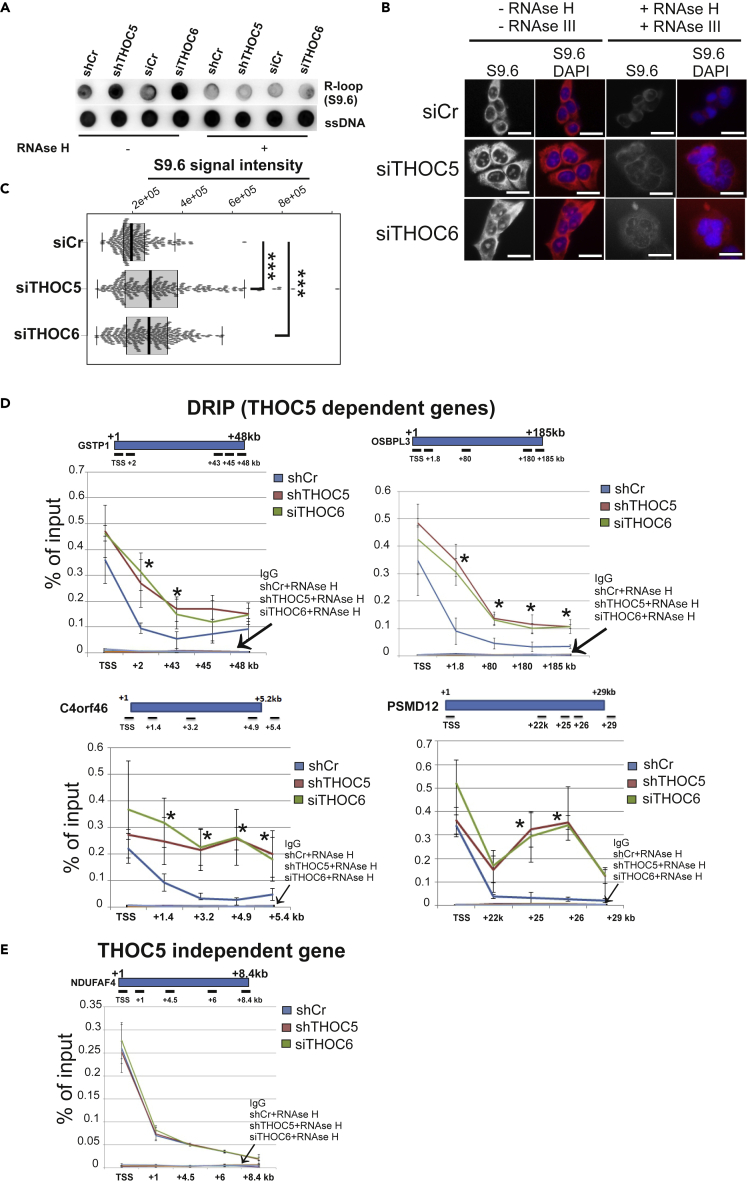
Depletion of THOC5 and THOC6 induces R-loop formation (A) Genomic DNA isolated from control (si, sh), THOC5- (shTHOC5) and THOC6 (siTHOC6)-depleted HEK293 cells was treated with/without RNAse H and subjected to Dot Blot analysis using R-loop-specific antibody (S9.6). ssDNA was used as loading control. (B and C) Representative images and quantification of nuclear RNA–DNA hybrid accumulation in THOC5- or THOC6-depleted HEK293 cells. Immunostaining with S9.6 monoclonal antibody and DAPI in the presence and absence of RNAse H and RNAse III in siC, siTHOC5, and siTHOC6 transfected cells. More than 50 cells per condition were counted in each of the three experiments. The median of the S9.6 signal intensity per nucleus is shown (n = 3). Scale bar, 200 μm. (∗∗∗) p < 10^−7^, Student t test, two-tailed. (D and E) DRIP: R-loop immunoprecipitation using genomic DNA isolated from control, THOC5- and THOC6-depleted cells were performed. R-loop profile of THOC5-dependent genes (*GSPT1*, *OSBPL3*, *C4ORF46*, and *PSMD12*) (B) and independent gene (*NDUFAF4*) (C) in the presence and absence of THOC5 or THOC6 are shown. Three to four independent experiments were performed, and the average signal of DRIP is shown as the mean value +/− SD. ∗p value <0.05.

It has been shown that transcription pausing/slowing may cause R-loop formation^27^ and that the yeast THO complex plays a key role in preventing this.^28^ Indeed, in our model, upon depletion of THOC5 and THOC6 the number of R-loops is increased as detected by anti R-loop antibody (S9.6) dot immunoblot (Figure 5A). As expected, RNAse H treatment drastically reduced the signal (Figure 5A). Application of anti-ssDNA antibody made no difference. We then assayed R-loops by immunofluorescence (IF) using the S9.6 antibody and also observed a nuclear accumulation of R-loops in THOC5- or THOC6-depleted cells (Figures 5B and 5C). The R-loop accumulation was drastically reduced upon RNAse H and III treatment, confirming the R-loop accumulation upon THOC5 or THOC6 depletion. We next mapped R-loop accumulation using DNA-RNA immunoprecipitation (DRIP) assay at the gene level. In the control cells, R-loops are mainly detected around the TSS (Figures 5D and 5E). Upon THOC5 or THOC6 depletion, R-loops clearly accumulated across the gene body of THOC5-dependent genes (Figure 5D), whereas gene body accumulation was not observed in THOC5 independent gene (Figure 5E). Here also, the R-loop signal is almost abolished upon treatment with RNAse H, suggesting the R-loops recruit THOC5 and THOC6 to the gene body.

### THOC6, but not THOC5, interacts directly with R-loops

**Figure 6:**
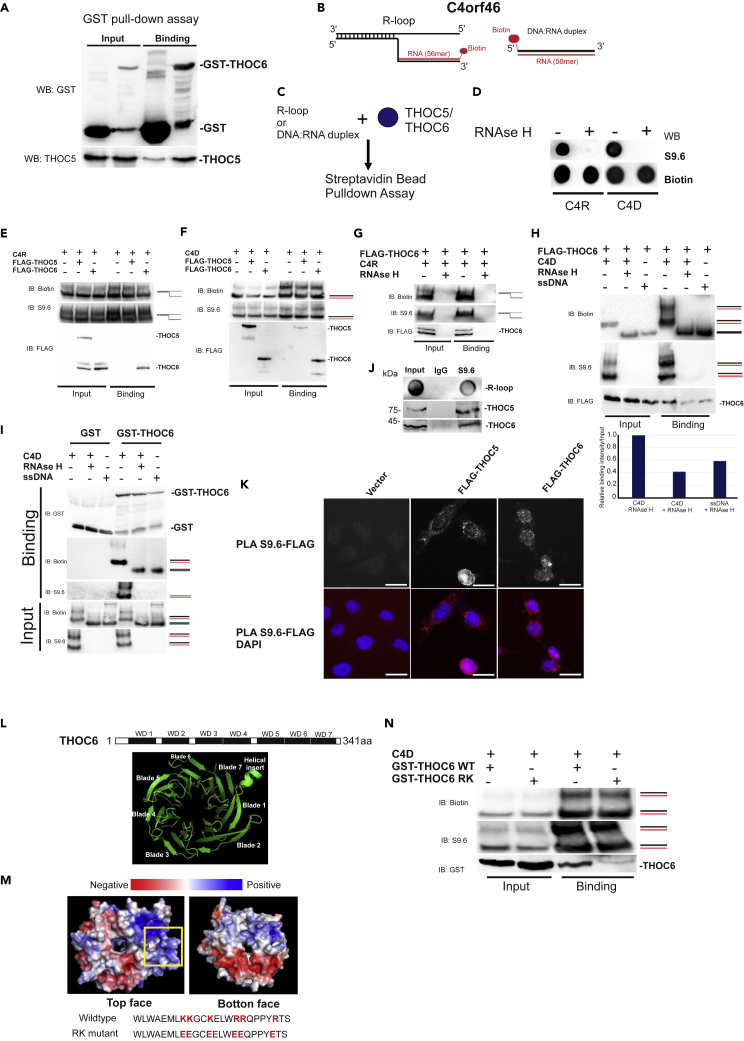
THOC6, but not THOC5, interacts directly with R-loops (A) GST pull-down assay was performed using recombinant GST and GST-THOC6 with purified FLAG-tagged THOC5. Both input and pull-down samples were detected using GST and THOC5-specific immunoblot. (B) RNA–DNA flap structure mimicking R loop as substrate or DNA-RNA heteroduplex derived from C4ORF46 intron 1 were used for subsequent interaction assays. (C) The DNA or RNA strand was labeled with Biotin at the 5′end. Biotin-labeled R-loops or DNA-RNA duplexes will be incubated with purified THOC5 or THOC6 and subjected to Streptavidin bead pull-down assay. (D) Synthesized R-loops or DNA-RNA duplexes were treated with/without RNAse H overnight at 37°C and subjected to Dot Blot analysis using R-loop (S9.6) and biotin-specific antibodies. (E and F) R-loops (C4R) or DNA-RNA duplexes (C4D) were incubated with purified FLAG-tagged THOC5 or THOC6 at 4°C overnight. R-loop-protein complexes were purified using Streptavidin Sepharose beads. Both input and pull-down samples were detected using R-loop (S9.6) and FLAG-specific immunoblot. Three independent experiments were performed, and a representative experiment is shown. (G and H) FLAG-tagged THOC6 was incubated with C4R (G) or C4D (H) in the presence and absence of RNAse H. R-loop or ssDNA were isolated using Streptavidin Sepharose beads. Both input and pull-down samples were detected using R-loop (S9.6) and FLAG-specific immunoblot. Three independent experiments were performed, and a representative experiment is shown. Immunoblot signal was quantified using ImageJ. (I) The GST pull-down assay was performed using recombinant GST and GST-THOC6 with DNA-RNA duplexes (C4D) in the presence and absence of RNAse H or with ssDNA. Both input and pull-down samples were detected using GST, R-loop (S9.6), and biotin-specific immunoblot. Three independent experiments were performed, and a representative experiment is shown. (J) Interaction between R-loops and THOC5/THOC6 in cells was examined by R-loop-specific co-IP study. Normal mouse IgG was used as a negative control. (K) The *in situ* interactions between R-loops and THOC5 or THOC6 were examined using PLA assay: FLAG-tagged THOC5 or THOC6 were overexpressed in HeLa cells for 2 days. Cells were then subjected to PLA assay using S9.6 and anti-FLAG antibody. Scale bar, 200 μm. (L) *In silico* prediction of THOC6 structure was generated using Phyre2. (M) 3 arginines and 3 lysines located in blade 2 of THOC6 were mutated to glutamic acid (RK mutant). *In silico* structure of THOC5 wild-type (WT) and RK mutant was generated using Phyre2. Electrostatic models are shown. (N) DNA-RNA duplexes C4D were incubated with recombinant GST-THOC6 WT and RK mutant. C4D–protein complexes were isolated using Streptavidin Sepharose beads. Both input and pull-down samples were detected using GST, R-loop (S9.6), and biotin-specific immunoblot. Three independent experiments were performed, and a representative experiment is shown. See also Figure S5.

To further examine the THOC5/THOC6-R-loop complex formation, we first performed a GST pull-sdown assay using a recombinant GST-THOC6 and a purified FLAG-tagged THOC5. As shown in Figure 6A, THOC5 formed a complex with THOC6 *in vitro*. Next, we examined which of the two directly interacts with R-loop. For that we synthesized a blunt-ended DNA-RNA heteroduplex (C4D and OSD) and a DNA-RNA hybrids with a 5′ ssDNA flap at one end as an R-loop mimetic structure (C4R) derived from the THOC5 target *C4ORF46* and *OSBPL3* (Figure 6B, Table S7). In addition, we labeled the ssDNA strand or ssRNA with biotin at the 5′end to detect and purify R-loops/DNA-RNA hybrids (Figure 6B). An R-loop-specific immunoblot gave a strong signal for the synthesized R-loop mimetic (C4R) or DNA-RNA heteroduplexes (C4D and OSD), respectively. The signals were completely abolished upon treatment with RNase H (Figure 6B). We next incubated the C4R, C4D, and OSD with purified FLAG-tagged THOC5 or THOC6 overnight at 4°C or at 37°C for 2 h. R-loop-protein complex was then isolated using streptavidin beads. Pull-down of R-loops was confirmed by Biotin- and S9.6 immunoblot (Figure 6C). Here, THOC6 clearly binds to both R-loop mimetic (C4R) and DNA-RNA duplex (C4D and OSD) at both 4°C and 37°C (Figures 6E and 6F (C4D); Figure S5A OSD), whereas THOC5 only slightly interacts with those. The R-loop-THOC6 interaction was completely abolished upon RNAse H treatment that digested the biotinylated RNA strands (Figure 6G). We further confirmed the R-loop-THOC6 interaction using an electrophoretic mobility shift assay (EMSA) (Figure S5B). We also examined the DNA-RNA duplex-THOC6 interaction in the presence and absence of RNAse H. RNAse H digested completely RNA strands, leaving behind biotinylated ssDNAs (Figure 6H). The binding of THOC6 to ssDNAs is clearly weaker, suggesting THOC6 preferentially interacts with DNA-RNA hybrid (Figure 6H). To confirm this observation, we incubated C4D (−/+ RNAse H) with GST-THOC6 and subsequently performed a GST pull-down assay. This again demonstrated that THOC6 binds more strongly to DNA-RNA hybrid than to ssDNA (Figure 6I).

To examine whether THOC5/THOC6 forms a complex with R-loops *in vivo*, we performed DNA-RNA hybrid IP. In addition, we overexpressed FLAG-tagged THOC5 or THOC6 and performed a proximity ligation assay using S9.6 antibody and FLAG antibody. As shown in Figure 6J, THOC5 and THOC6 were co-immunoprecipitated with R-loops, suggesting that they form a complex in cells. We confirmed this interaction by proximity ligation assay (PLA, Figure 6K).

How does THOC6 interact with R-loops? We predicted the 3D structure of THOC6 using Phyre2^29^ to identify potential R-loop binding domains. Structural data generated by Phyre2 revealed that THOC6 is a seven β-propeller protein in which the seventh blade is formed of three anti-parallel strands from the C-terminal region of the domain and is completed with a strand originating from the N-terminus of the domain (Figure 6L). Notably, electrostatic surface of the top face reveals a prominent basic patch that covers blade 1, 2, and 7 and the helical insert region (Figure 6M, blue region), suggesting that these regions possibly interact with R-loops. To test this possibility, we mutated 3 arginines and 3 lysines located in blade 2 into glutamic acid (RK mutant, Figure 6M, yellow box). Notably, the RK mutant does not alter the predicted seven β-propeller structure (Figure S5B). We next incubated GST-THOC6 wild type and the RK mutant with R-loops and subsequently purified the R-loop complex using streptavidin beads. Although THOC5 still binds to the THOC6 RK mutant (Figure S5C), binding of the RK mutant to the R-loop is much weaker compared with wild-type THOC6, suggesting that the positively charged region of blade 2 is involved in interaction with R-loops (Figure 6N).

### DDX5/17 resolve R-loops in THOC5 target genes

**Figure 7:**
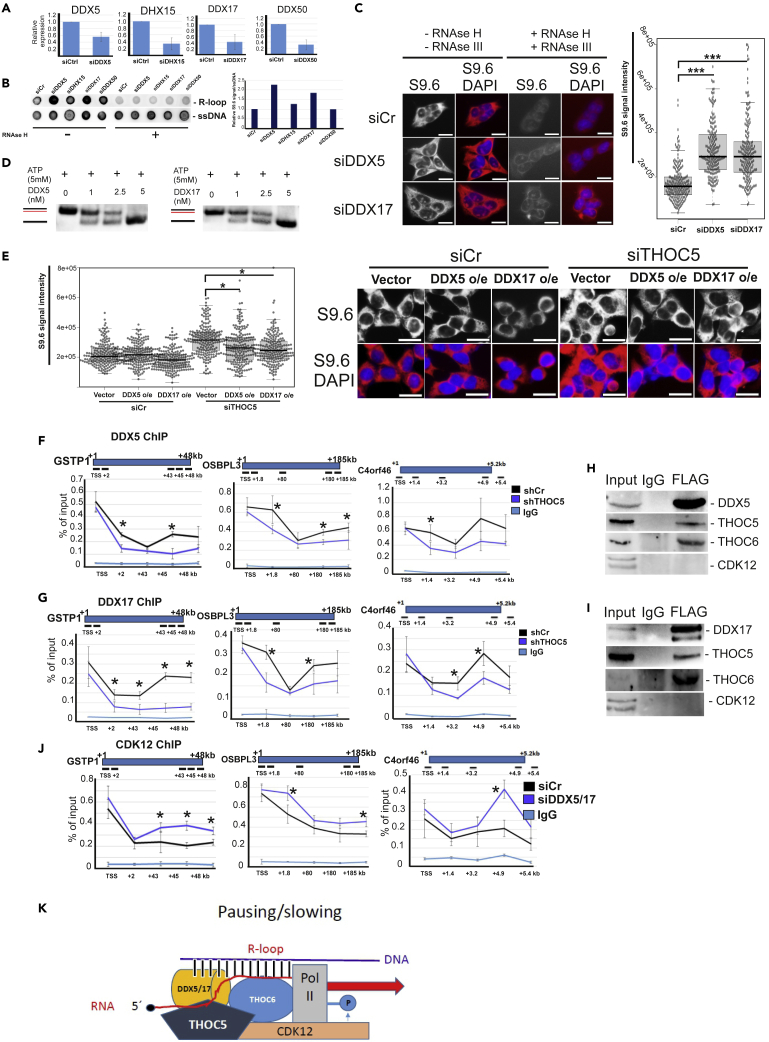
THOC5 recruits DDX5 and DXX17 during transcription elongation (A) DDX5, DHX15, DDX17, and DDX50 were depleted using siRNA. Knockdown efficiency was confirmed by qRT-PCR. Three independent experiments were performed, and the average signal of qRT-PCR is shown as the mean value +/−SD. (B) Genomic (g)DNAs were isolated from control and DDX-depleted cells. gDNA with/without RNAse H treatment were subjected to Dot Blot analysis using R-loop-specific antibody (S9.6). ssDNA was used as loading control. Dot blot signal was quantified using ImageJ. (C) Representative images and quantification of nuclear RNA–DNA hybrid accumulation in DDX5- or DDX17-depleted HEK293 cells. Immunostaining with S9.6 monoclonal antibody and DAPI in the presence and absence of RNAse H and RNAse III in siCr, siDDX5, and siDDX17 transfected cells. More than 50 cells per condition were counted in each of the three experiments. The median of the S9.6 signal intensity per nucleus is shown (n = 3). Scale bar, 200 μm. (∗∗∗) p < 10^−20^, Student t test, two-tailed. (D) R-loop unwinding assay in the presence of increasing DDX5 or DDX17. (E) Representative images and quantification of nuclear RNA–DNA hybrid accumulation in THOC5-depleted HEK293 cells transfected with empty vector (Vector) or FLAG-tagged DDX5 (DDX5 o/e) or DDX17 (DDX17 o/e). More than 50 cells per condition were counted in each of the three experiments. The median of the S9.6 signal intensity per nucleus is shown (n = 3). Scale bar, 200 μm. (∗) p < 10^−4^, Student t test, two-tailed. (F and G) FLAG-tagged DDX5 (F) and DDX17 (G) were overexpressed in control and THOC5-depleted cells. Cells were fixed in 1% PFA and supplied for ChIP assay using FLAG M2 antibody. The recruitment profile of DDX5 and DDX17 to *GSPT1*, *OSBPL3*, and *C4ORF46* genes in the control and THOC5-depleted cells is shown. (H and I) FLAG-tagged DDX5 (H) and DDX17 (I) were overexpressed in HEK293 cells. Co-IP studies using FLAG M2 antibody were performed to examine the interaction with THOC5, THOC6, and CDK12. Both input and pull-down samples were detected using FLAG-, THOC5-, THOC6-, and CDK12-specific immunoblot. (J) DDX5 and DDX17 were depleted using siRNA. Control and DDX5/17 double knockdown cells were fixed in 1% (v/v) PFA and subjected to CDK12 ChIP assay. The recruitment profiles of CDK12 to *GSPT1*, *OSBPL3*, and *C4ORF46* gene in control and DDX5/17-depleted cells are shown. Three independent experiments were performed, and the average signal of ChIP is shown as the mean value +/−SD. ∗: p value <0.05. (K) Novel role of the THOC5/THOC6 complex during transcription elongation. When transcription elongation rate is low and R-loops are formed, the THOC5/THOC6 complex recruits CTD kinase CDK12, which phosphorylates the CTD domain of RNA polymerase II at serine 2 and enhances the elongation. At the same time, the THOC5/THOC6 complex also recruits DNA-RNA helicases DDX5 and DDX17 to resolve the R-loops, thus preventing transcription elongation impairment and premature termination. See also Figure S6.

The interactome analysis using chromatin-associated THOC5 as a bait indicated that 4 RNA helicases, namely, DDX5, DDX17, DDX50, and DHX15, interact with THOC5 (Table S4). To determine which of these helicases are involved in R-loop formation, each helicase was depleted by siRNA (Figure 7A), and the accumulation of R-loops in whole genome was examined using an R-loop-specific antibody S9.6. Upon depletion of DDX5 and DDX17, but not DDX50 and DXH15, the number of R-loops was increased (Figure 7B). We also observed an R-loop accumulation at the cellular level using R-loop specific IF in DDX5- or DDX17-depleted cells (Figure 7C). We then tested their RNA–DNA unwinding activity *in vitro* by isolating DDX5 and DDX17 as GST fusion proteins and incubated them with C4D DNA-RNA duplexes. We observed that both DDX5 and DDX17 could unwind this substrate in a protein-concentration-dependent manner (Figure 7D). Next, we examined whether DDX5 and DDX17 possess an *in vivo* unwinding ability in our cell system by overexpressing them in THOC5-depleted cells. Overexpression of DDX5 or DDX17 suppressed the R-loop accumulation in THOC5-depleted cells (Figure 7E), suggesting that they suppress the R-loop accumulation *in vivo*, consistent with the observation *in vitro* (Figure 7D).

We then examined whether DDX5 and DDX17 are recruited to the THOC5 target genes. Both DDXs are recruited near the promoter region and are increasingly recruited toward the TES (Figures 7F and 7G). When THOC5 was depleted, the recruitment of DDX5/17 to the gene body, but not to the promoter, was strongly decreased, whereas R-loops clearly accumulated across the gene body of THOC5-dependent genes upon DDX5 or DDX17 depletion (Figure S6A). These data suggest that THOC5 is required for the recruitment of DDX5/17 during transcription elongation. We next examined whether THOC5/THOC6 complex regulates the enzyme activity of DDX5/17 by performing unwinding assays in the presence and absence of THOC5/THOC6. Our data suggest that THOC5/THOC6 did not modulate the unwinding activity of DDX5/17 (Figure S6B). Interestingly, both DDX5 and DDX17 bound to the THOC5/THOC6 complex, but not to CDK12 (Figures 7H and 7I). To examine whether DDX5/17 and CDK12 collaborate to resolve R-loops and enhance transcription elongation, we depleted both DDX5 and DDX17 and performed a ChIP assay of CDK12. CDK12 is increasingly recruited to the recruitment sites of DDX5/17 in DDX5/17-depleted cells (Figure 7J).

## Discussion

THOC5 is an important gene product in stem cell biology and has been shown to be post-translationally regulated by stem cell ligands (CXCL12), oxidative stress, and the downstream actions of oncogenes. This set of facts indicate the later development of this protein is critical in higher organisms, although the precise role for the protein has not been elucidated. Here mechanistic details on THOC5 and THOC6 function are elucidated. Complementing the finding by Yan et al. who state that R-loops have a role in cell fate determination, memory, and plasticity,^30^ here we show a link from THOC5 to R-loops that may explain the exquisite sensitivity of stem cells to THOC5 depletion.

Previously, we have shown that THOC5 plays a role in the 3′processing of inducible genes, such as *c-MYC*.^8^ In this study, by applying Nanopore RNA sequence technology, we show here that the depletion of THOC5 influences not only the 3′processing of inducible genes, but approximately 50%–60% of all mRNAs. This altered 3′processing leads to shorter mRNAs but has little effect on the efficiency of mRNA export. However, it is not presently known whether all these shorter mRNAs are translated.

We have previously shown that the most rapidly induced IEGs such as *c-FOS*, *IER2* or *ZFP36* with fast elongation rates are processed and exported in the absence of THOC5.^8^ In this study, we show that THOC5 depletion decreased elongation rates (Figures 2E–2G). Elongation rates of THOC5-dependent genes are 2-fold lower than those of THOC5 independent genes (Figure 2K), suggesting that THOC5 is selectively recruited to slow transcribed genes. These data raise the question of whether elongation rates affect poly(A) usages and whether THOC5 is more required in cells expressing slow Pol II than in cells expressing fast Pol II. Our Nanopore direct RNA-seq data suggest that fast Pol II provokes distal 3′cleavage, whereas slow Pol II provokes proximal cleavage (Figures 3B and 3C). In these cell systems, we observed a higher recruitment of THOC5 to its target genes in slow Pol II cells compared with that in fast Pol II cells (Figure 3D). The interactome analysis of chromatin-associated THOC5 and results of the ChIP assay suggest that the THOC5/THOC6 complex directly supports transcription elongation of genes with slow elongation rate by recruiting CDK12 that phosphorylates Ser2 of the Pol II carboxy-terminal domain (CTD).^26^ The depletion of THOC5 or THOC6 reduces CDK12 recruitment to the gene body (Figure 4G). Subsequently, released Pol II molecules accumulated downstream of TSS (Figures 2G and 2I). Furthermore, nanopore direct RNA-seq data suggested that THOC6 and CDK12 depletion also provokes proximal 3′end cleavage similar to that observed in THOC5-depleted cells (Figures 4A and 4B). Notably, 3′end cleavage of >50% of THOC5-dependent mRNAs are also regulated by THOC6 and CDK12 (Figure 4C). In line with our data, it has been shown that the inhibition or depletion of CDK12 leads to premature termination and transcript shortening of a subset of genes.^31^ These data suggest that THOC5 influences 3′end cleavage via modulating the elongation rate.

It has been reported that THOC5 is also involved in digestion of left-over RNA strands after cleavage to avoid R-loop formation.^32^ Interestingly, our interactome analysis data suggest that THOC5 interacts with DEAD box helicase DDX5, which resolves R-loops at RNA polymerase II transcription termination sites and also interacts with XRN2 (5′-3′ Exoribonuclease 2), a transcription termination factor (Table S4) that degrades the uncapped residual RNA from the 5′ to the 3′ until it reaches the RNA Pol II unit.^27^^,^^33^^,^^34^ Our ChIP and DRIP data revealed that DDX5 helicase was increasingly recruited toward the 3′end of THOC5 target genes (Figure 7F) and the depletion of DDX5 induced R-loop accumulation across these genes (Figure S6A), suggesting that THOC5 may be also involved in transcription termination in a DDX5- and XRN2-dependent manner.

Is the THO complex one functional unit? Recent data from our and other groups suggest that each member of the THO complex plays different roles during transcription/export of mRNA.^10^^,^^12^^,^^16^^,^^35^^,^^36^ Interestingly, Puhringer et al. have recently reported that THOC1/2/3 still form a stable monomeric complex in the absence of THOC5/6/7, whereas THOC5 and THOC6 are required for the tetrameric complex formation of the THO–UAP56/DDX39B complex.^37^ In line with these data, we show in this study that THOC5 and THOC6 form a subcomplex and participate in transcription elongation, whereas the depletion of THOC1, THOC2, or THOC3, but not THOC5 and THOC6, inhibits the bulk mRNA export,^12^^,^^17^ suggesting that members of THO complex form subcomplexes and participate in multiple steps of co-transcriptional mRNA export.

Is it the THO complex or THOC5 that is critical in R-loop biology? In agreement with previous data in yeast and *C. elegans*,^11^^,^^38^ we also observed R-loop accumulation when members of the THO complex are depleted (Figure 5A). We show in this study that THOC5 and THOC6 play a key role in removing harmful R-loops during transcription elongation by recruiting the DNA-RNA helicases DDX5 and DDX17 (Figures 7F and 7G). Incidentally mouse models show that CDK12, DDX5, and DDX17 are essential genes during development, like THOC5.^39^^,^^40^ Also, DDX5 and its paralog DDX17 are known to play a role in R-loop resolution.^33^^,^^34^ Notably, the depletion of DDX5/17 increased the recruitment of CDK12 to the gene body, suggesting that R-loop formation during transcription elongation might slow down the Pol II (Figure 7J). Here we show why these 4 proteins when knocked out may elicit a common phenotype. Our *in vitro* data suggested that THOC5/THOC6 complex binds to R-loops in general (Figure 6). It raises the question of what determines the target gene selectivity. THOC5 selectively targets gene with slow elongation rates (Figure 2K), and interactome analysis data suggested that THOC5 interaction partners in slow Pol II cells are enriched in DNA damage/DNA repair signaling (Figure S7). Notably, harmful R-loop accumulation during transcription elongation induces DNA damage response. Furthermore, THOC5 per se is a substrate of ATM kinase that plays a key role in DNA damage response,^41^^,^^42^ suggesting that DNA damage sensors may play a role in defining the specificity of THOC5/THOC6 on genes with slow elongation rates during transcription elongation. We are currently investigating whether the modulation of elongation rate directly influences co-transcriptional R-loop formation.

Although a number of factors have been proposed to resolve or prevent R-loops, helicase UAP56/DDX39B is the only factor known to act co-transcriptionally.^10^^,^^43^ Notably, the protective role of UAP56 is more evident for longer and highly transcribed genes. In this study, we propose a second factor, namely THOC5/THOC6 that functions co-transcriptionally to resolve R-loops via recruiting DNA-RNA helicase DDX5/17 to a subset of genes with a slow elongation rate (Figure 7K). Although THOC6 was found to interact directly with R-loops (Figure 6), it does not have a typical RNA binding domain. These data suggest that the THOC5/THOC6 complex may transiently interact with and transfer DDX5/17 to R-loops. We show that both DDX5 and DDX17 possess an RNA–DNA unwinding activity (Figures 7D and 7E). In line with our data, it has been reported that DDX5 is able to unwind RNA–DNA hybrid *in vitro* and *in vivo,*^33^^,^^34^^,^^44^ whereas DDX17 can unwind double-stranded RNAs.^45^^,^^46^ Data from our and other groups also raise the question of why cells need different DDXs to resolve harmful R-loops accumulated during transcription elongation in a context-dependent manner. Thus, comparison of helicase activities may help to better understand the possible coexistence of different mechanisms to prevent R-loops.

In summary, our data propose a novel role of the THOC5/THOC6 complex during transcription elongation. When transcription elongation of a subset of genes slows significantly, R-loops are formed. R-loops recruit THOC6 that forms a complex with THOC5. The THOC5/THOC6 complex recruits both CTD kinase CDK12 and DNA-RNA helicases to resolve R-loops and enhance elongation that further supports the 3′end cleavage at the distal sites. Reduced transcriptional elongation rate results in early embryonic lethality in mice.^47^ Thus, THOC5 may ensure this does not happen to allow stem cell survival. We now have mechanistic detail to understand signal transduction-mediated regulation of THOC5, potential targeting to induce death in malignant cells and also the molecular biology of R-loop regulation in health and disease.

### Limitations of the study

In this study, we show that THOC5 depletion altered 3′processing leads to shorter mRNAs but has little effect on the efficiency of mRNA export. However, it is unclear whether all these shorter mRNAs are translated. Our data also do not rule out whether the modulation of elongation rate directly influences co-transcriptional R-loop formation.

## STAR★Methods

### Key resources table

**Table:** 

REAGENT or RESOURCE	SOURCE	IDENTIFIER
**Antibodies**		

Mouse anti-THOC5	Generated and provided by Mancini et al.^48^	NA
Rabbit anti-THOC5	Bethyl	Cat#A302-120A; RRID:AB_1604293
Goat anti-Beta Actin	Santa Cruz	Cat#sc-1616; RRID:AB_630836
Mouse anti-THOC1	Abcam	Cat#ab487; RRID:AB_304696
Rabbit anti-THOC2	Novus	Cat#NBP1-92500; RRID:AB_11020045
Mouse anti-THOC3	LSBio	Cat#LS-C31568; RRID:AB_2287257
Mouse anti-THOC6	LSBio	Cat#LS-B4536; RRID:AB_10798780
Rabbit anti-THOC7	Sigma	Cat#HPA044143; RRID:AB_10794892
Rabbit anti-UAP56	Proteintech	Cat#14798-1-AP; RRID:AB_2061854
Rabbit anti-CHTOP	Invitrogen	Cat#PA5-44307; RRID:AB_2576376
Rabbit anti-NXF1	Abcam	Cat#ab50609; RRID:AB_881770
Rabbit anti-WTAP	Abcam	Cat#ab195380; RRID:AB_2868572
Rabbit anti-Histon H3	Cell Signaling	Cat#9715S; RRID:AB_331563
Mouse anti-ssDNA	Novus	Cat#NBP2-29849
Mouse anti-R-loops (S9.6)	Kerafast	Cat#ENH001; RRID:AB_2687463
Rabbit anti-CDK12	Cell Signaling	Cat#11973S; RRID:AB_2715688
Rabbit anti-CDK12	Proteintech	Cat#26816-1-AP; RRID:AB_2880645
Rabbit anti-Pol II	Abcam	Cat#ab26721; RRID:AB_777726
Rabbit anti-FLAG	Proteintech	Cat#20543-1-AP; RRID:AB_11232216
Rabbit anti-FLAG	Sigma	Cat#SAB1306078
Red ANTI-FLAG® M2 Affinity Gel	Sigma	Cat#F2426; RRID:AB_2616449

**Bacterial and virus strains**		

BL21 (DE3) strain	NEB	Cat#C2527H

**Chemicals, peptides, and recombinant proteins**		

DMEM High-Glucose medium	PAN Biotech	P04-05550
Fetal bovine serum	Thermo Fisher	10270098
Penicillin-streptomycin-glutamine	Gibco	10378016
Ampicillin sodium salt	SigmaAldrich	A0166
IPTG	Gold Biotechnology	I2481C5
4-thiouridine	Glentham Life Sciences	GN6085
Chloroform	Thermo Fisher Scientific	43685
Chloroform/isoamyl alcohol	Sigma-Aldrich	C0549
TRIzol	Thermo Fisher	15596026
Ethanol	VWR	24105
2-mercaptoethanol	Sigma-Aldrich	M3148-25ML
SDS pellets	Sigma-Aldrich	75746
MTSEA biotin-XX linker	Biotium	BT90066
μMACS Streptavidin Kit	Miltenyi	130-074-101
5,6-dichlorobenzimidazole 1-β-D-ribofuranoside	Sigma-Aldrich	D1916

**Deposited data**		

Nanopore- and ChIP sequencing	NA	Accession number GSE173374
Interactome analysis	NA	Accession number PXD035092

**Experimental models: Cell lines**		

HEK293 cells	ATCC	Cat#CRL-1573
HEK293 expressing fast Pol II mutant (N792D/E1126G)	Kindly provided by Fong et al.^49^	NA
HEK293 expressing slow Pol II mutant (N792D/R749H)	Kindly provided by Fong et al.^49^	NA

**Oligonucleotides**		

Primer sequences	See Table S6	NA
siRNA sequences	See Table S5	NA
R-loop sequences	See Table S7	

**Software and algorithms**		

Seqmonk	Babraham institute	NA
Galaxy workflow for sequencing analysis	www.usegalaxy.org	NA
DRB_TT-seq	Described by Gregersen et al.^23^	NA
Mascot	Matrix Sciences	NA
Ingenuity® Pathway Analysis (IPA)	Qiagen	NA
NanoFilt	Described by De Coster et al.^50^	NA

### Resource availability

#### Lead contact

Further information and requests for resources and reagents should be directed to and will be fulfilled by the lead contact, Doan Duy Hai Tran (Tran.Doan@mh-hannover.de).

#### Materials availability

All the nucleotide sequences of the plasmids used are available from the lead contact without restriction.

### Experimental model and subject details

Experimental model for this study was derived from HEK293, a cell line exhibiting epithelial morphology that was isolated from the kidney of a human embryo. Cells were cultured in DMEM (PAN Biotech). DMEM were supplemented with 10% bovine growth serum (Thermo Fisher) and penicillin-streptomycin-glutamine (Gibco). Cells were grown at 37°C in standard cell culture incubators. All cells were routinely tested and confirmed to be free of mycoplasma contamination. A full list of cell lines used in the paper is included in the key resources table.

### Method details

#### Protein purification

Coding sequences of THOC5, THOC6, DDX5 and DDX17 were cloned into pcDNA3.1+/C-(K)-DYK and purchased by Genscript (NJ, USA). THOC5, THOC6, DDX5 and DDX7 were transiently overexpressed in HEK293 and purified after two days using anti-FLAG M2 affinity gel (Sigma, MA, USA).

For GST pulldown assay, the coding sequences of FLAG tagged THOC6, DDX5 and DDX17 were subcloned into pGEX-4T1 vector. Protein expression was induced in BL21 (DE3) strain by adding IPTG and proteins were purified using Glutathione Sepharose (GE Healthcare, IL, USA). GST fusion proteins and GST tag were separated using TEV protease. TEV protease was then removed using NEBExpress® Ni-NTA Magnetic Beads (NEB).

#### Cell culture and lentiviral transduction

HEK293 cells were grown and transduced with control shRNA (shCr) and shRNA targeting THOC5 (shTHOC5) as previously described.^19^ si- and shRNA sequences are listed in Table S5. HEK293 cells expressing a Dox inducible RNA Pol II mutant^49^ were maintained with 200 μg/mL hygromycin B and 6.5 μg/mL blasticidin. These cells were transduced with control shRNA and shRNA targeting THOC5. After one to three days, control- and THOC5 depleted cells were treated with 2.0 μg/mL doxycycline for 12–16 h and with 2.5 μg/mL α-amanitin for a further 42 h, at which time all cell lines were viable, and endogenous Pol II was inactive. All cell lines were free of mycoplasma contamination.

#### RNA isolation

Cytoplasmic RNA was isolated as previously described.^8^ PolyA + mRNA was isolated from cytoplasmic RNA using the NEBNext® PolyA + mRNA Magnetic Isolation Module (New England Biolabs, MA, USA).

#### RNA sequencing and data processing

PolyA + RNA (500 ng) were used for the nanopore direct RNA sequencing. A library was prepared using a direct RNA sequencing kit (SQK-RNA002, Oxford Nanopore Technologies Ltd.) according to the manufacturer’s protocol. Libraries were sequenced on R9.4 flow cells for 48 h. Base calling was performed using Guppy (Oxford Nanopore). Basecalled data were aligned to human reference genome GRCh38 using minimap2.^51^

#### 3′end cleavage analysis

For the identification of 3′cleavage sites the tool NanoFilt^50^ (Filtering and trimming of long read sequencing data) was used to trim the mRNA sequences from the 5′end to a uniform length so that the last 200 nucleotides (python get_read_ends.py --bases_from_end 200 reads.fastq.gz | gzip > last_200_bp.fastq.gz) are retained. Trimmed reads were aligned to the GRCh38 or GRCh37 human genome references using minimap2. The quantification of 3′ cleavage was performed with Seqmonk using the human polyA database.^52^ Genes that contain more than two annotated polyadenylation sites (PAS) and more than 5 reads at distal PAS sites were selected for downstream analysis. The ratios between the proximal- and distal cleavage sites were calculated.

#### Chromatin immune precipitation (ChIP)

ChIP experiments were performed as previously described.^8^ Briefly, aliquots of 5 × 106 cells were fixed in 1% (v/v) paraformaldehyde for 5 min and subsequently quenched in 125 mM glycine. The nuclear fraction was isolated and resuspended in sonication buffer (0.1% SDS, 50 mM TrisHCl, pH 8.0, 0.2 mM EDTA, 1x Protease inhibitor cocktail (Sigma)). Chromatin was sheared into 500 bp DNA fragments using Covaris AFA™ (Adaptive Focused Acoustics, Woburn, MA, USA) technology according to the manufacturer’s instructions. After shearing, NaCl and NP-40 were added to final concentration of 150 mM and 1%, respectively. Aliquots of extracts were immunoprecipitated using Protein G Agarose-PLUS (Santa Cruz Biotechnology Inc., TX, USA) pre-coated with anti-RNA polymerase II (Abcam, Cambridge, GB), THOC5, CDK12, FLAG M2 antibodies or control IgG. Following 4 h rotation at 4°C, the beads were washed three times in RIPA buffer (150 mM NaCl, 1% NP-40, 0.1% SDS, 50 mM TrisHCl at pH 8), and two times in wash buffer (500 mM NaCl, 1% NP-40, 0.1% SDS, 100 mM TrisHCl at pH 8). Cross-links were reversed for 8 h at 65°C (250 mM NaCl) in the presence of RNAse A. Following proteinase K digestion at 55°C for 1 h, the bound DNA fraction was isolated using NucleoSpin Extract II (Macherey-Nagel, Dueren, Germany).

#### ChIP sequencing and data analysis

5 ng of ChIP derived DNA were subjected to library preparation using TruSeq ChIP Library Preparation Kit (Illumina, CA, USA). Libraries were prepared and indexed according to manufacturer’s protocol. Indexed libraries were pooled and sequenced on an Illumina HiSeq 2500 (Illumina). FASTQ files were generated by CASAVA (v1.8.2). Galaxy workflow (www.usegalaxy.org) was used for subsequent data analysis. Reads were mapped to the human reference genome (GRCh38) using Bowtie2 (Galaxy Version 2.3.4.1). PCR duplicates were detected and removed using MarkDuplicates (Picard). Metagene analysis was performed using deepTools 2.0.^53^ MACS peak calling with Seqmonk was applied to identify peaks enriched in each dataset. Raw data were deposited in GSE173374.

#### DRB/TT_chem_-seq

DRB/TTchem-seq was performed as described previously.^23^ The treatment protocol was depicted in Figure 2D. Briefly, one 10-cm dish of HEK293 cells at 70% confluency was prepared for each time point, experimental sample, or control. Cells were treated with 100 μM 5,6-dichlorobenzimidazole (DRB) for 3.5 h for each time point after release. The DRB inhibition was released with three washes in 10 mL of PBS pre-warmed to 37 °C. Fresh medium containing 1 mM 4SU was directly added to the cells after PBS washes as indicated in Figure 2D. RNAs were isolated using TRIzol reagent and fragmented in 166 mM NaOH on ice for 20 min. To biotinylate 4SU-RNAs 3 μL of biotin buffer (833 mM Tris-HCl, pH 7.4, and 83.3 mM EDTA) and 50 μL of 0.1 mg/mL MTSEA biotin-XX linker (Biotium, CA, USA) were added to the 200 μL of fragmented RNA and incubated at RT for 30 min in the dark. Biotinylated RNAs were isolated by adding equal volume of phenol/chloroform/isoamyl alcohol (25:24:1 (v/v/vol)) and subsequently precipitated in NaCl/isopropanol. Biotinylated RNA fragments were then separated from non-biotinylated fragments using μMACS streptavidin Micro-Beads (Miltenyi Biotec, Bergisch Gladbach, Germany). The incorporation of 4SU was tested using dot blot analysis (Figure S1A). Biotinylated RNAs were analyzed on a Bioanalyzer (Figure S1B) before being subjected to a paired-end sequencing (Illumina, SD, USA).

To measure the progression of Pol II molecules into the gene body we applied a pipeline described by Gregersen et al. for calling RNA Pol II transcription wave peak positions and elongation rates from DRB/TT-seq time-series data. The pipeline first created a set of genomic intervals representing the TSS region (−2 kb: +120 kb) of non-overlapping protein coding genes 60–300 kb in width from standard chromosomes (4591 genes). The read coverage was then calculated and normalized to the sequencing depth.

To calculate the elongation rates the module “wave peak calling for single gene” was applied. This module filtered out poorly expressed genes (e.g., total base-pair coverage over the −2 kb: +120 kb region <100 rpm) and genes with a wave peak <2 kb in the first (e.g., 10 min) sample to reduce noise from the TSS region.

#### Isolation of nucleoplasmic and chromatin associated fractions

HEK293 cells (10^7^) were lysed by cytoplasmic lysis buffer (50 mM TrisHCl, pH 8.0, 140 mM NaCl, 1.5 mM MgCl_2_, 0.5% NP-40, RNase inhibitor, 1 mM DTT). After centrifugation (800xg for 2 min) supernatant (cytoplasmic fraction) was removed and the pellet was resuspended in 60 μL NUN1 buffer (20 mM Tris-HCl (pH 7.9), 75 mM NaCl, 0.5 mM EDTA and 50% (v/v) glycerol). Six-hundred μl of ice-cold NUN2 buffer (20 mM HEPES-KOH (pH 7.6), 300 mM NaCl, 0.2 mM EDTA, 7.5 mM MgCl_2_, 1% (v/v) NP-40 and 1 M urea) was then added to the suspensions, vortexed and further incubated on ice for 15 min. After centrifugation, supernatant was collected as nucleoplasmic fraction. The pellet was incubated with Turbo DNAse I (Thermo Fisher Scientific, MA, USA) in high salt buffer (containing 500 mM NaCl, 1x protease inhibitor cocktail (Sigma)) for 20 min at 37°C (chromatin associated fraction). For co-immunoprecipitation study the chromatin associated fraction was diluted 1:3 with 50 mM Tris-HCL, pH 8 buffer containing 0.5% NP-40.

#### Co-immunoprecipitation (Co-IP) using chromatin associated fraction

Chromatin associated extracts were diluted in 50 mM Tris-HCl pH 8 to a concentration of 150 mM NaCL and then NP-40 was added to a concentration of 0.5% (v/v). The diluted extracts were immunoprecipitated by Protein G Agarose-PLUS with mouse monoclonal anti-THOC5, FLAG M2 antibodies or control IgG in the presence of RNAse A. Following 8 h rotation at 4°C, the beads were washed five times. Input and binding samples were supplied for immunoblot or interactome analysis.

#### Analysis of chromatin associated THOC5 interacting proteins

Precipitated THOC5 protein complexes and control IgGs were subjected to electrophoresis on 10% (w/v) polyacrylamide gels and stained with simply Blue Coomassie safe stain (Thermo Fisher Scientific, MA, USA). Precipitated THOC5 protein complexes and control IgGs were subjected to electrophoresis on 10% (w/v) polyacrylamide gels and stained with simply Blue Coomassie safe stain (Thermo Fisher Scientific). Protein bands were excised and destained with repeated incubation in 200 mM ammonium bicarbonate, 40% (v/v) acetonitrile. Gel pieces were dried with 3 washes in acetonitrile and then trypsinized (trypsin resuspended in 100 mM ammonium bicarbonate, 5% (v/v) acetonitrile) overnight at 37°C. Peptides were extracted from the gel pieces by incubation in 50% (v/v) acetonitrile, 0.1% (v/v) formic acid, and peptides were desiccated and resuspended in 3% (v/v) acetonitrile, 0.1% (v/v) formic acid, 20 mM citric acid, pH 2.7. For each analysis, 10% of the peptide sample was loaded onto a nanoACQUITY UPLC Symmetry C18 Trap (5 μm, 180 μm × 20 mm) (Waters Corporation, MA, USA), and flow was set to 15 μL/min of 3% (v/v) acetonitrile, 0.1% (v/v) formic acid, and 20 mM citric acid for 5 min. Analytical separation of the peptides was performed using a nanoACQUITY UPLC BEH C18 column (1.7 μm, 75 μm × 250 mm) (Waters Corporation). Briefly, peptides were separated over a 91-min solvent gradient from 3% (v/v) acetonitrile, 0.1% (v/v) formic acid to 40% (v/v) acetonitrile, 0.1% (v/v) formic acid on-line to an LTQ Orbitrap Velos (Thermo Fisher Scientific). Data were acquired using an information-dependent acquisition method where, for each cycle one full MS scan of *m*/*z* 300–1700 was acquired in the Orbitrap at a resolution of 60,000 at m/z 400 with an automatic gain control target of 106. Each full scan was followed by the selection of the 20 most intense ions; CID (collision-induced dissociation) and MS/MS analysis was performed in the LTQ Orbitrap Velos instrument (Thermo Fisher Scientific). Selected ions were excluded from further analysis for 60s. Ions with an unassigned charge or a charge of +1 were rejected.

Data were analyzed using Mascot (Matrix Sciences, IL, USA) software; the parameters were: Uniprot database, trypsin with up to 1 missed cleavage allowed, variable modifications oxidized methionine, phosphorylated serine, threonine, and tyrosine modifications permitted and peptide tolerance of 0.025 and 0.03 Da for MS/MS tolerance.

Proteins which were detected in both control IgG and precipitated THOC5 complexes were discarded. Then, THOC5 interaction partners which had Mascot >30 were further selected for Ingenuity® Pathway Analysis (IPA).

#### Immunoblot procedures

Details of immunoblotting have been described previously.^54^ Antibodies used in this study are listed in the key resources table.

#### Semi-quantitative RT-PCR and qRT-PCR analysis

Semi-quantitative RT-PCR and qRT-PCR were performed as previously described.^55^ Primer pairs for each PCR are described in Table S6.

#### DNA-RNA dot blot

Genomic DNA was isolated by using the QIAmp DNA Mini kit (Qiagen, Hilden, Germany, 51304). Genomic DNA (500 ng) was treated with 10U of RNase H (New England Biolabs, M0297S) for 8 h at 37°C before loading on the dot blot and transferred onto an N+ membrane (Macherey-Nagel, Dueren, Germany). Following UV crosslinking at 1200 μJ, membranes were blocked in 5% milk/PBS-Tween (0.05% Tween 20) and incubated overnight with S9.6 (1:1000) or ssDNA (Novus, FL, USA) antibody. After the last wash, the membrane was incubated for 1 h at RT with secondary antibody (goat anti mouse horseradish peroxidase). After washing three times for 10 min with PBS-Tween (0.05% Tween 20), the membrane was briefly dried and incubated with Super-Signal West Femto chemiluminescent substrate (Thermo Fisher Scientific) for 2 min. The chemiluminescent signal was detected by exposure of the membrane to ImageQuant™ LAS 4000 (GE Healthcare, IL, USA).

#### GST pull-down assay

Recombinant GST, GST-THOC6 WT and GST-THOC6 mutant were incubated with purified THOC5, 50 pmol of R-loop or ssDNA for 2 h rotation at 4°C. After washing, input and bound fractions were analyzed by THOC5, R-loop (S9.6 antibody) or Biotin specific immunoblot.

#### Streptavidin pull-down assay

Biotinylated R-loop or ssDNA were incubated with purified FLAG-THOC5, THOC6 WT or THOC6 RK-mutant for 16 h rotation at 4°C or for 2 h at 37°C. Input and bound fractions were analyzed by FLAG M2, R-loop (S9.6 antibody) or Biotin specific immunoblot.

#### DNA-RNA immunoprecipitation (DRIP)

DRIP was performed as previously described.^56^ Briefly, cells (1 × 107) were washed with ice-cold PBS and resuspended in 1.6 mL of TE buffer. Then, 50 μL of 20% (w/v) SDS and 5 μL of 20 mg/mL proteinase K were added and further incubated at 37 °C for 12 h. DNA lysate was poured directly into the Maxtract phase-lock gel tube (Qiagen) and 1 vol (1.6 mL) of phenol/chloroform/isoamyl alcohol (25:24:1) was added. DNA was precipitated with NaAc. The DNA pellet was air-dried and sobulized in TE buffer. Extracted genomic DNA was digested using a cocktail of restriction enzymes (BsrGI, EcoRI, HindIII, SspI and XbaI) at 37 °C overnight. Digested DNA was then isolated using phenol-chloroform precipitation. 8 μg of extracted DNA were diluted in DRIP binding buffer (10 mM sodium phosphate, pH 7, 140 mM NaCl and 0.05% (v/v) Triton X-100). Twenty micrograms of the S9.6 antibody were added to the diluted DNA and further incubated for 14–17 h at 4 °C. Then protein G PLUS-agarose (Santa Cruz Biotechnology Inc.) was added and incubated for 2 h at 4 °C. Precipitates were isolated using NucleoSpin Extract II (Macherey-Nagel).

#### Helicase unwinding assays

Unwinding assays were performed in MOPS buffer (25 mM MOPS (morpholinepropanesulfonic acid), pH 7.0, 60 mM KCl, 0.2% Tween 20, 2 mM DTT, 5 mM ATP, 5 mM MgCl_2_). DDX5 or DDX17 and labeled DNA-RNA duplexes (100 nM) substrates were incubated in MOPS buffer for 60 min at 37°C, followed by deproteinization in one-fifth volume of stop buffer (20 mM Tris-Cl, pH 7.5, and 2 mg/mL proteinase K) for 30 min at 37°C. The resolution of R-loop was confirmed by immunoblotting using Biotin antibody.

#### R-loop immunofluorescence (IF)

Cells were fixed in ice-cold methanol for 10 min and then incubated in staining buffer (TBST with 0.1% BSA) for 10 min at RT. Enzymatic treatments were done in RNAse H buffer with 1:100 dilutions of RNAse H (NEB) and ShortCut RNase III for 4h at 37°C. Samples were subsequently washed by incubating with staining buffer for 10 min with rocking. For primary immunolabeling, samples were incubated in staining buffer with 1:200 dilutions of mouse S9.6 antibody (Kerafast) for 2h at RT with rocking. Samples were then washed once with staining buffer and incubated with 1:200 dilutions of secondary anti-mouse TRITC conjugate (Sigma) and counterstained with DAPI. Nikon NIS elements D 3.0 Software was used to quantify the fluorescence. Only the foci and nuclear S9.6 signal intensity were quantified.

#### R-loop co-immunoprecipitation (co-IP)

Nuclear extracts in RIPA buffer containing 1x Protease inhibitor cocktail (Sigma) were sonicated using Covaris AFA™ (Adaptive Focused Acoustics, Woburn, MA, USA) technology according to the manufacturer’s instructions. After centrifugation (13,000 rpm for 10 min at 4°C), extracts were immunoprecipitated using Protein G Agarose-PLUS (Santa Cruz Biotechnology Inc., TX, USA) pre-coated with S9.6 antibody or control IgG. Following 4 h rotation at 4°C, the beads were washed three times in RIPA buffer. Precipitated R-loops or bound proteins were visualized using immunoblot. For S9.6 dot blot, aliquots of input, IgG and S9.6 IP were treated with proteinase K before subsequent R-loop purification.

#### Proximity ligation assay (PLA)

HeLa cells were transfected with FLAG tagged THOC5, THOC6 and empty vector for two days. Cells were fixed in 4% PFA for 15 min at RT and permeabilized with 0.2% Triton X-100 for 5 min. Samples were blocked in blocking buffer of Proximity ligation assay for 1h at 37°C. After blocking, samples were incubated with S9.6 and FLAG antibody (rabbit, Sigma) (1:200 dilution in antibody dilution buffer) for 2h at 37°C. After 2x washing with wash buffer A, the samples were incubated with pre-mixed Duolink PLA plus and minus probes for 1 h at 37°C. The subsequent steps in proximal ligation assay were carried out using the Duolink® PLA Fluorescence kit (Sigma) according to the manufacturer’s instructions.

#### Electrophoretic mobility shift assay

200 nM of biotin labeled R-loop mimic substrate (C4R) were incubated with 10 μM THOC6 in the binding buffer containing 20 mM Tris pH 7.5, 2 mM DTT, 12 mM MgCl2, 100 mM NaCl, and 10% glycerol for 30 min at RT. The complexes were separated by native PAGE and visualized by Biotin immunoblot.

### Quantification and statistical analysis

Cell experiments were performed in a minimum of three independent experiments (n ≥ 3). Data are displayed as the mean with error bars reported as +/− SD. The statistical significance was determined by the Student´s test (two-sided) (Excel) for Figures 2A, 2B, 2K, 4G, 4H, 5C–5E and 7C, 7E, 7F, 7G, 7J. The exact value and description of n were described in the corresponding figure ligands. A p value of less than 0.05 was considered as statistically significant difference.

## Data Availability

• Nanopore- and ChIP sequencing data have been deposited at GEO and are publicly available as of the date of publication. Accession numbers are listed in the key resources table. Microscopy data reported in this paper will be shared by the lead contact upon request. • Interactome analysis data have been deposited at ProteomeXchange and are publicly available as of the date of publication.

## Supplemental information

Document S1. Figures S1–S7 and Tables S4–S7

Table S1. List of 626 THOC5 dependent- and 213 THOC5 independent genes, related to Figure 1

Table S2. Elongation rates of THOC5 dependent- and independent genes, related to Figure 2

Table S3. Slow Pol II THOC5 interaction partners, related to Figure 3
