# Supplementary material for: THOC5 complexes with DDX5, DDX17, and CDK12 to regulate R loop structures and transcription elongation rate
Source: iScience. 2022 Dec 9;26(1):105784. doi: 10.1016/j.isci.2022.105784 (PMC9800341; doi:10.1016/j.isci.2022.105784)
Supplement: Document S1. Figures S1–S7 and Tables S4–S7 [file mmc1.pdf]

iScience, Volume *26*

Supplemental information

THOC5 complexes with DDX5, DDX17, and CDK12 to regulate R loop structures and transcription elongation rate

Mareike Polenkowski, Aldrige Bernardus Allister, Sebastian Burbano de Lara, Andrew Pierce, Bethany Geary, Omar El Bounkari, Lutz Wiehlmann, Andrea Hoffmann, Anthony D. Whetton, Teruko Tamura, and Doan Duy Hai Tran

Supplemetal figures

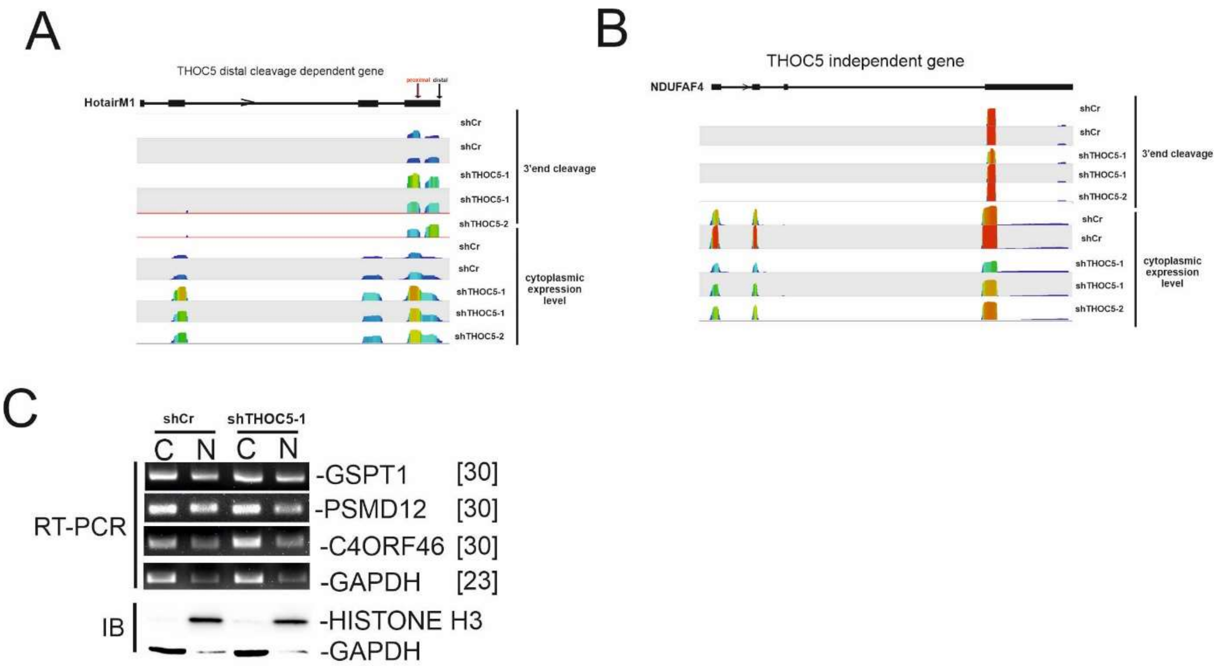

Figure S1: The read coverage of THOC5 independent- and distal cleavage dependent genes, related to Figure 1. (A,B) Wiggle plot of *HotairM1* and *NDUFAF4* gene: Seqmonk was used to quantitate and visualize sequencing data. Peaks in the wiggle plot represent the normalized RNA-seq read coverage. TSS: transcription start site. TES: transcription end site. (C) mRNA export assay: Nuclear and cytoplasmic RNAs were isolated from control- and THOC5 depleted cells and applied for *GSPT1*, *PSMD12*, *C4ORF46* and *GAPDH*-specific RT–PCR using proximal primers (Table S6). As control for fractionation, aliquots of protein extracts from each sample were supplied for, Histone H3 (nuclear fraction), and GAPDH (cytoplasmic fraction)-specific immunoblot (immunoblot). []: number of PCR cycles.

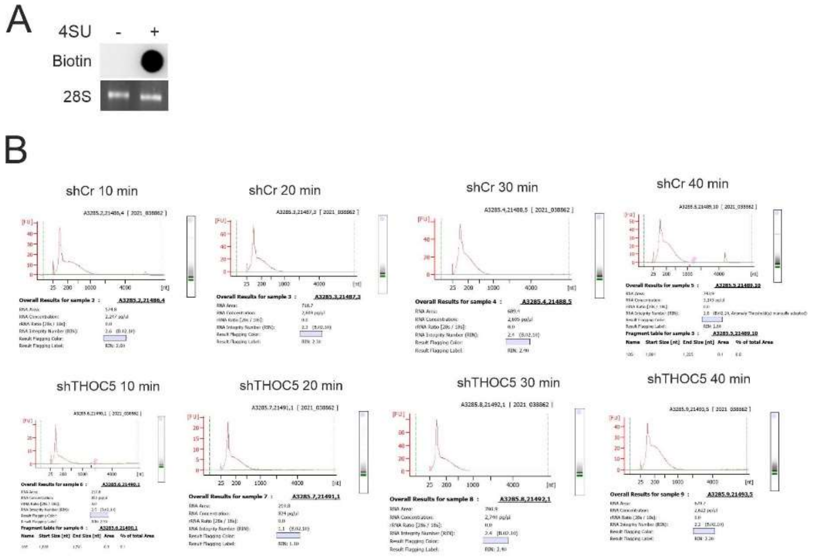

Figure S2: Sample preparation for DRB/TT_{chem}-seq, related to Figure 2. (A) Check of 4SU incorporation: HEK293 cells were treated with 1mM 4SU for 15min. RNAs were isolated. Control for 4SU incorporation into RNA by slot blot of biotinylated RNA using HRP-conjugated streptavidin (HRP- streptavidin). Staining of 28S RNAs serves as a loading control. (B) Bioanalyzer results of RNA after biotinylation, fragmentation and streptavidin pull-down.

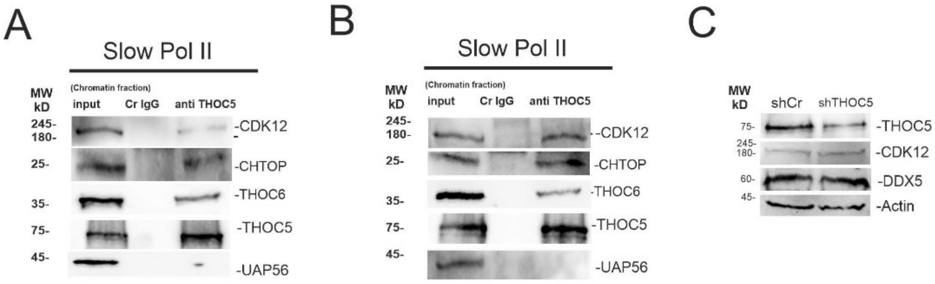

Figure S3: **Validation of THOC5 interactome using co-IP study, related to Figure 3.** (A,B) Replicates of endogenous THOC5 co-IP study in slow Pol II cells. (C) THOC5 was depleted using shTHOC5 RNAs. Cell lysates were subjected to THOC5, CDK12 and DDX5 specific immunoblotting.

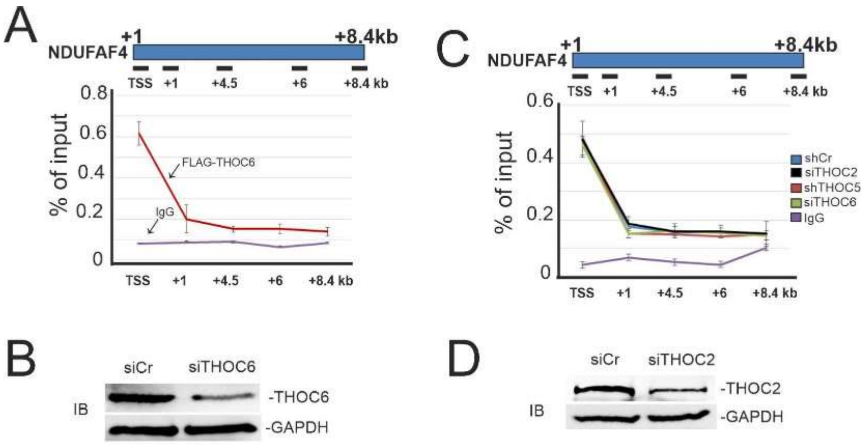

Figure S4: The recruitment profile of THOC6 and CDK12 to THOC5 independent gene, related to Figure 4. (A) FLAG tagged THOC6 was overexpressed in HEK293 cells. Cells were fixed in 1% (v/v) PFA and supplied for ChIP assay using FLAG M2 mouse monoclonal antibody. Mouse IgG was used as a control. The recruitment profile of THOC6 to NDUFAF4 is shown. Three independent experiments were performed and the average signal of ChIP is shown as the mean value +/- SD. (B) THOC6 was depleted using siRNAs. The depletion efficiency was confirmed by immunoblot. (C) THOC5 were depleted in HEK293 cells by lentivirus (sh) while THOC2 or THOC6 were downregulated by siRNAs. Cells were fixed in 1% PFA and CDK2 ChIP assay performed. The recruitment profile of CDK12 to NDUFAF4 in the presence and absence of THOC5 or THOC6 is shown. (D) THOC2 was depleted using siRNAs. The depletion efficiency was confirmed by immunoblot.

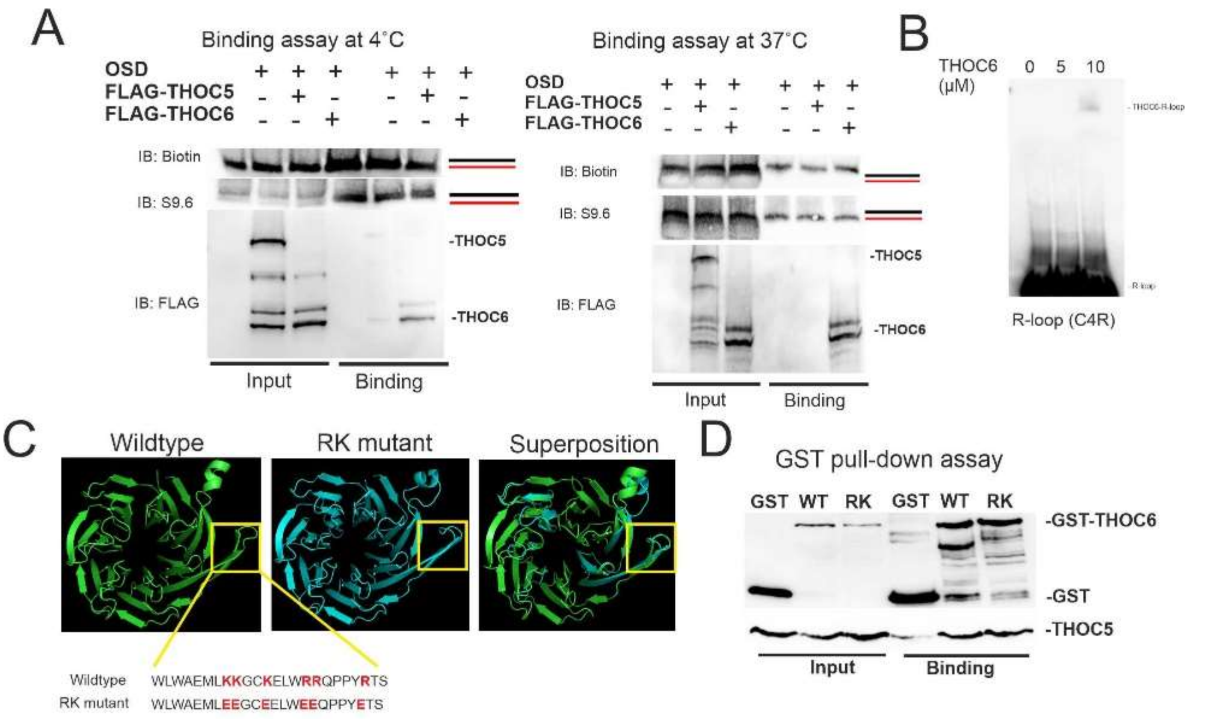

Figure S5: THOC5/6-DNA-RNA duplex interaction, related to Figure 6. (A) DNA-RNA hybrids OSD were incubated with purified FLAG tagged THOC5 or THOC6 at 4 ºC overnight or 37 ºC for 4h. DNA-RNA hybrids-protein complexes were purified using Streptavidin Sepharose beads. Both input and pulldown samples were detected using R-loop (S9.6) and FLAG specific immunoblot. (B) The R-loop-THOC5 interaction was confirmed using an electrophoretic mobility shift assay (EMSA). (C) Superposition of THOC6 wildtype and RK mutant. (D) Interaction of between THOC6 WT or RK mutant with THOC5 was examined by GST pull-down assay.

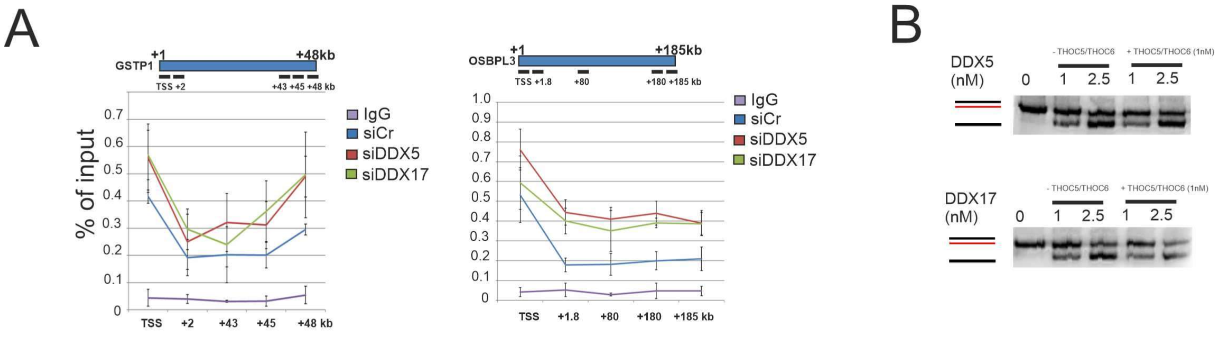

Figure S6: DRIP analysis and helicase activity of DDX5 and DDX17, related to Figure 7. (A) DRIP: R-loop immunoprecipitation using genomic DNA isolated from control, DDX5 and DDX17 depleted cells were performed. R-loop profile of THOC5 dependent genes *GSPT1* and *OSBPL3* in the presence and absence of DDX5 or DDX17 are shown. Three to four independent experiments were performed and the average signal of DRIP is shown as the mean value +/-

SD. (B) R-loop unwinding assay in the presence of increasing DDX5 or DDX17 (-/+ purified THOC5/THOC6).

Top 4 molecular and cellular functions
of THOC5 interaction partners in fast and slow Pol II cells

Slow Pol II

Name	p-value	#Molecules
RNA Post-Transcriptional Modification	1,24E-02 - 1,81E-12	20
Cell Death and Survival	1,86E-02 - 2,34E-06	50
Cell Cycle	2,34E-02 - 4,53E-06	34
DNA Replication, Recombination, and Repair	1,86E-02 - 4,53E-06	23

Fast Pol II

Name	p-value	#Molecules
Cell Death and Survival	5,71E-07 - 4,56E-12	93
Cellular Movement	2,00E-09 - 2,00E-09	65
Cellular Assembly and Organization	7,67E-08 - 3,74E-09	56
Cellular Function and Maintenance	2,63E-08 - 3,74E-09	52

Figure S7: Functional pathway analysis of THOC5 interaction partners, related to Figure 3 and Discussion. Ingenuity® Pathway Analysis (IPA) was performed to identify functional enrichment observed within the proteins that interact with THOC5 in either in slow Pol II cells or fast Pol II cells. The significance (p-values) for the function enrichment was calculated using the right-tailed Fisher's Exact Test (Kramer et al. 2014) are shown.

Supplemental Tables

Table S4: Proteins involved in RNA processing identified as interacting with THOC5 in slow Pol II cells, related to Figure 3.

**Gene**	**Description**
AFF2	AF4/FMR2 family member 2
CDK12	cyclin dependent kinase 12
DDX5	DEAD-box helicase 5
DDX17	DEAD-box helicase 17
DDX50	DExD-box helicase 50
DHX15	DEAH-box helicase 15
EXOSC3	exosome component 3

NOLC1	nucleolar and coiled-body phosphoprotein 1
NOP58	NOP58 ribonucleoprotein
SNW1	SNW domain containing 1
SOX6	SRY-box 6
SRRM1	serine and arginine repetitive matrix 1
SRSF2	serine and arginine rich splicing factor 2
SRSF3	serine and arginine rich splicing factor 3
THRAP3	thyroid hormone receptor associated protein 3
TRA2A	transformer 2 alpha homolog
U2AF1/U2AF1L5	U2 small nuclear RNA auxiliary factor 1
XRN2	5'-3' exoribonuclease 2
YTHDC1	YTH domain containing 1
ZNF326	zinc finger protein 326

Table S5: si- and shRNA sequences, related to STAR Methods.

Name	Sequence
shTHOC5-1	ATCAAACTGATACTGATTG
shTHOC5-2	ATCAAACTGATACTGATTG
siTHOC6	GCCAAAGAGGAAAGUAAGA
siCDK12	ATCATTTTTGTGGTTTTGAAA
siDDX5	UGCAUGGCCUUCCAAUAAA
siTHOC2	TGGCTTGTTCTTAATATTAGT
siDDX17	CAAAGAAUCUUAAGGGUUU + ACUGCUCUGUGCUUUCAAA
siDDX15	UUACCUGGCUGCUUUAAAU
siDDX50	UUGAAAGACUCCAAAGAAA

Table S6: Primer table, related to STAR Methods.

Gene	Acession No	Forward	Reverse	ChIP	qRT-PCR	RT-PCR
PSMD12 (TSS)	ID: 5718	AACTTCCGGTGTGGGTGACGAGT	TGTAGTCCACCTCCATCTTGACGAT	x	x	
PSMD12 (+22kb)		ACCACAATGGAGTTGATGCGTTGGT	TGTCATCTCTATGACAGATTCTTACATGT	x	x	
PSMD12 (+25kb/proximal)		TCCGAAGCCTTTCTCTCAAATCT	CTCTTTGGCTATGAGATGCGTA	x	x	x

PSMD12 (+26kb/distal)		AGCTGAGTGTTACTAACTCTTAAGA	AGAGAAAGGCTTCGGACTCCT	x	x	x
PSMD12 (+29kb)		TCACACCTCCCTCATTGTCAACA	GAAGTATTATGGTAGTATAATGGTGGATAC	x	x	
C4ORF46 (TSS)	ID: 201725	AGTGGTTAAAAGACAGTTGGTGTCGGT	AAGATGCTGCAGAGGCGTCTGAAGA	x	x	
C4ORF46 (+1.4kb)		AGTGTTCACAGGTCAATGCCCATGT	ACACTAGAGTCTCAGCTCAACAAGATGA	x	x	
C4ORF46 (+3.2kb/proximal)		GATCTTAGTGACAGTGTTCTATTTTGCA	TCTCCATATCTACAAAGAATTAAGATGTTGT	x	x	x
C4ORF46 (+4.9kb/distal)		TGATACCAAGATGAGAAGATGGCT	GAGAAGTGTGACAAGACGAAGGA	x	x	x
C4ORF46 (+5.4kb)		CTATTGAGTTATGTCAGCTTAGGACAA	TGGTAATGGACCGTTTACTACGT	x	x	
GSPT1 (TSS)	ID: 2935	TGACTAGTCAAACAAACGGTGAGTC	GGTGCAGATTGCAAAGCTGATTTC	x	x	
GSPT1 (+2kb)		CCCAGCCGAGAGAGCGTCTTC	GCGTACACAATGCTAAGGGTAAAACAGA	x	x	
GSPT1 (+43kb)		TCTAGGTAAGACCATTGCAATTGGA	ACAGTGGCATAGCAGAGCTCT	x	x	
GSPT1 (+45kb)		TGCTAACCATGTTTTTGATGAACCA	AGAGAAATCACAATCTAAAAGAGGCT	x	x	
GSPT1 (+48kb)		TCACCTCCATTTCCTCCACATGCA	TGAGATCTAGGTATTAACCTGCTGTCTA	x	x	
OSBPL3 (TSS)	ID: 26031	GAGGAGGACAGAGCAAGCCT	GTTCCTAAACCACCTCGCAAAG	x	x	
OSBPL3 (+1.8kb)		GTCATGGAATCCGCTGGCTTC	ATGGCCACTTGCAGACAGACT	x	x	
OSBPL3 (+80kb)		ATGGGCATTTATAGTGGGTTGTATGCA	AATGGTCACTGGCTGACTTCTTTGTTAC	x	x	
OSBPL3 (+180kb)		AACCTCTGCCTTCCAGTTCCA	TCACGCCTGTAATCTCGGTAC	x	x	
OSBPL3 (+185kb)		CCTCTGCATTGTACTAATCTGTTACTCA	CCACTTCTGATCACTGCATTCCCCA	x	x	
NDUFAF4 (TSS)	ID: 29078	CTCACTTGTGCGCATGCTC	ACTAATCTGCTCTCGCAGGA	x	x	
NDUFAF4 (+1kb)		GCTGCTGTCACGGACTTGATACA	AGTGCTGAGATTACAGGCTTGAGCT	x	x	
NDUFAF4 (+4.5kb)		TGTGCTTGGAGTGGAAGGAAGATCT	TCTCATAACTGCAGTGAACTCTAACCA	x	x	
NDUFAF4 (+6kb)		CAGCTGTTAGGGTGGCATCA	TGAGCCATAGTTTAATAGTACTTTTGCCA	x	x	
NDUFAF4 (+8.4kb)		TGCTTCCGAGGGAAGATAGGCA	CACTTAGTCCATTTTTCTGTTCTCAGCA	x	x	
GAPDH	NM_002046.7	TGTTGCCATCAATGACCCCTT	CTCCACGACGTACTCAGCG		x	
DDX5	NM_001320595.2	AGAGACTTATACTCATGCTCTTGGCT	AGAGACTTATACTCATGCTCTTGGCT		x	
DDX17	NM_006386.5	CAGGTGGCCGATGACTATGG	TCGAATCTGGGGACCTTTAGG		x	
DHX15	NM_001358.3	TATCAGTGCTGGATTACCACCT	GAATGCGTTGAATGTGCTGAG		x	
DDX50	NM_024045.2	CATCCAGTGTCATTGGTCTCAG	GCAGCCACATTGGTTGCCAC		x	

Table S7: Oligos used in the generation of DNA:RNA hybrid structures, related to STAR Methods. Complementary sequences are colour coded:

C4-ssDNA-Forward	5'TAATCCCAGCTACTAAGGAGGCTGAGGCAGGAGAATCACTTGAACCCGGGAGGCGGAGGTTGCAGTGAACCAAGATCGCGCCATTGCACTCCAGCCTGGG 3'
C4-ssDNA-Reverse	5'CCCAGGCTGGAGTGCAATGGCGCGATCTTGGTTCACTGCAACCTCCGCCTCCCGGGCTCAAGTGATTCTCCTGCCTCAGCCTCCTTAGTAGCTGGGATTA
C4-ssDNA	5'CCCGGGAGGCGGAGGTTGCAGTGAACCAAGATCGCGCCATTGCACTCCAGCCTGGG 3'
C4-ssRNA	5'CCCAGGCTGGAGTGCAATGGCGCGATCTTGGTTCACTGCAACCTCCGCCTCCCGGG 3'
OS-ssDNA	5'CGCCCTCCCGCCCCCTCGGGGCCCCGCCCCTGGGCCTCTCAGGCCCCTCCCCTGCGCTCC 3'
OS-ssRNA	5'GGAGCGCAGGGGAGGGGCCTGAGAGGCCCAGGGGCGGGGCCCCGAGGGGGCGGGAGGGCG 3'

2 step annealing procedure for generation of different DNA:RNA hybrid structures:

Substrate	Step 1		Step 2	
	Oligos	Temp	Oligos	Temp
Migratable 5' Flap (C4R R-loop)	(A) C4-ssDNA-Forward + C4-ssRNA	100ºC 5min, slowly cooled down to RT	Mix A + C4-ssDNA-Reverse	37ºC, 1h
C4D (DNA-RNA duplex)	C4-ssDNA + C4-ssRNA	100ºC 5min, slowly cooled down to RT	-	-
OS4D (DNA-RNA duplex)	OS-ssDNA + OS-ssRNA		-	-
